# Assessing metastatic potential of breast cancer cells based on EGFR dynamics

**DOI:** 10.1038/s41598-018-37625-0

**Published:** 2019-03-04

**Authors:** Yen-Liang Liu, Chao-Kai Chou, Mirae Kim, Rohan Vasisht, Yu-An Kuo, Phyllis Ang, Cong Liu, Evan P. Perillo, Yu-An Chen, Katherine Blocher, Hannah Horng, Yuan-I Chen, Duc Trung Nguyen, Thomas E. Yankeelov, Mien-Chie Hung, Andrew K. Dunn, Hsin-Chih Yeh

**Affiliations:** 1 Department of Biomedical Engineering, The University of Texas at Austin, Austin, TX USA; 2 Department of Molecular and Cellular Oncology, The University of Texas MD Anderson Cancer Center, Houston, TX USA; 3 Department of Electrical and Computer Engineering, The University of Texas at Austin, Austin, TX USA; 4 Department of Bioengineering, The University of Maryland, College Park, MD USA; 5 Institute for Computational Engineering and Sciences, The University of Texas, Austin, TX USA; 6 Department of Diagnostic Medicine, Dell Medical School, The University of Texas at Austin, Austin, TX USA; 7 Department of Oncology, Dell Medical School, The University of Texas at Austin, Austin, TX USA; 8 Livestrong Cancer Institutes, The University of Texas at Austin, Austin, Texas USA; 9 Center for Molecular Medicine and Graduate Institute of Cancer Biology, China Medical University, Taichung, Taiwan; 10 Texas Materials Institute, The University of Texas at Austin, Austin, TX USA

## Abstract

Derailed transmembrane receptor trafficking could be a hallmark of tumorigenesis and increased tumor invasiveness, but receptor dynamics have not been used to differentiate metastatic cancer cells from less invasive ones. Using single-particle tracking techniques, we  developed a phenotyping asssay named Transmembrane Receptor Dynamics (TReD), studied the dynamics of epidermal growth factor receptor (EGFR) in seven breast epithelial cell lines and developed a phenotyping assay named Transmembrane Receptor Dynamics (TReD). Here we show a clear evidence that increased EGFR diffusivity and enlarged EGFR confinement size in the plasma membrane (PM) are correlated with the enhanced metastatic potential in these cell lines. By comparing the TReD results with the gene expression profiles, we found a clear negative correlation between the EGFR diffusivities and the breast cancer luminal differentiation scores (r = −0.75). Upon the induction of epithelial-mesenchymal transition (EMT), EGFR diffusivity significantly increased for the non-tumorigenic MCF10A (99%) and the non-invasive MCF7 (56%) cells, but not for the highly metastatic MDA-MB-231 cell. We believe that the reorganization of actin filaments during EMT modified the PM structures, causing the receptor dynamics to change. TReD can thus serve as a new biophysical marker to probe the metastatic potential of cancer cells and even to monitor the transition of metastasis.

## Introduction

Receptor tyrosine kinases (RTKs) control many cell decision-making functions such as proliferation, survival, and movement. It has been shown that the important activities of RTKs are deregulated in most human cancers^1^. One form of the deregulation is the compromised spatial control and trafficking of RTKs^2^. While mounting evidence suggested that the derailed spatial regulation of RTKs could be a hallmark of tumorigenesis or even increased tumor invasiveness, very few reports studied the relationship between RTK dynamics and cancer cell behaviors. Grove’s group studied the dynamics of EphA2 receptors and showed the clustering of EphA2 receptors is coupled with the increased invasiveness of cancer cells^3^. While this work demonstrated that subtle changes in the spatial organization of transmembrane receptors can lead to malignant cell behaviors, there is no attempt to use the receptor dynamics as a biophysical phenotyping method for cancer cells. By measuring the dynamics of RTKs, we believe it is possible not only to differentiate cancer cells with distinct malignant states but also monitor the transition from pre-malignant state to metastatic state.

Traditional phenotyping assays are based on molecular analyses of genomic, epigenetic, transcriptomic or proteomic biomarkers, which often suffer from the problems of high cost and large variation in today’s single-cell analysis. To provide a multifaceted description of cancer cells, researchers have recently begun to explore physical properties of cancer cells (e.g., morphology^4^, viscoelasticity^5^, shear rheology^6^, and motility^7^), with a hope to find an alternative way to quickly and precisely identify highly invasive cancer subtypes^8,9^. These physical science approaches have revealed dramatic differences in mechanics, migration, and adhesion between MCF10A (non-tumorigenic) and MDA-MB-231 (highly invasive) breast cell lines^8^. However, most of these physical interrogation methods have one or more of the following issues (SI Fig. S1): the need to physically touch the adherent cells using a special tool (e.g., a tip of atomic force microscopy (AFM)^5^ or a micropipette aspiration device^10^), low information content (e.g., only one physical property, viscoelasticity, is measured in AFM), and low throughput (e.g., only one cell can be interrogated at a time by optical tweezers^11^). Currently, there is no physical interrogation technique that overcomes all of the above issues.

To address this challenge, we have developed a new biophysical phenotyping method termed **T**ransmembrane **Re**ceptor **D**ynamics (TReD), and showed that changes of TReD can be a signature of increased invasiveness. Our TReD phenotyping assay relies on an optical interrogation method (single-particle tracking of fluorescently tagged EGFRs) which not only avoids any physical manipulation of the cells but provides rich information about the receptors (e.g., transition probabilities between different diffusive states) and the microenvironment where the receptors are contained (e.g., confinement size). Here we demonstrate that EGFR dynamics, as an example of TReD, can be used to differentiate breast cell lines with distinct metastatic potential and monitor the epithelial-mesenchymal transition in the benign cell line. While our results agree well with the previous reports, our TReD assay is substantially easier than the current methods.

## Results

### TReD assay on the breast cell lines

**Figure 1:**
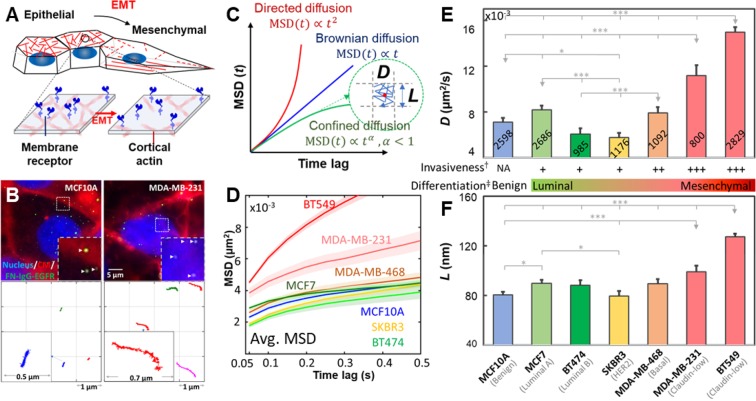
TReD, a new biophysical phenotyping method to assess metastasis. (**A**) Schematic shows the hypothetical effect of EMT on the EGFR dynamics. (**B**) Images of living cells and fluorescently labeled EGFRs (FN-IgG-EGFRs). The representative FN-IgG-EGFR trajectories shown in the lower panel were derived from the EGFRs pinpointed by the arrowheads. Individual trajectories were color-coded for identification. These trajectories were reconstructed from 60 seconds time-series images acquired in MCF10A and MDA-MB-231 cells. The insets are the zoom-in of trajectories. (**C**) The diffusivity of EGFR (*D*) and the linear size of the EGFR confinement (*L*) can be extracted from trajectories using a modified MSD fitting algorithm. (**D**) Averaged-MSD curves from 800–2,800 trajectories acquired in the seven breast epithelial cell lines. The solid line and the ribbon represent mean value and standard error of the mean, respectively. (**E**,**F**) Characterization of EGFR diffusivity (*D*) and compartment size (*L*) among these seven breast cell lines. More invasive breast cancer cell lines exhibit higher EGFR diffusivities and larger compartment sizes. The number of trajectories collected from each cell line is shown on each bar. Statistical comparison was performed using unpaired t-test., where the asterisk represents statistical significance: ***p < 0.001, **p < 0.01, *p < 0.05. The error bar represents the standard error of the mean. ^†^I*n vitro* invasiveness was derived from Lin’s report^22^. ^‡^Luminal differentiation scores of breast cancer cells were derived from the Perou^23^ and the Partanen^24^ reports.

To elucidate the connections among EGFR dynamics, PM compartmentalization, and invasiveness of cancer cells, we have performed the TReD assay on EGFRs in seven breast epithelial cell lines: MCF10A, MCF7, BT474, SKBR3, MDA-MB-468, MDA-MB-231, and BT549. EGFR was chosen in this study because its signaling network is compromised in many forms of human cancers^1,12^. In addition, EGFR can directly interact with actins^13,14^, altering not only the EGF-EGFR binding affinity but also the EGFR dimerization kinetics^15,16^. We believe EGFR dynamics are coupled to the signaling networks through the local actin environment of the cancer cells, and changes in cancer cell behaviors, such as epithelial-mesenchymal transition, can alter the EGFR dynamics (Fig. 1A). Trajectories of 800-2,800 single EGFR complexes (termed FN-IgG-EGFR, as EGFRs were tagged with anti-EGFR IgG antibody-conjugated fluorescent nanoparticles, Fig. 1B)were analyzed per cell line using a modified mean-squared displacement (MSD) fitting algorithm^17,18^, generating an averaged EGFR diffusivity (*D*) and a size of the linear confinement (*L*)^19^ for each cell line (Fig. 1C,D). Based on the molecular classification of breast carcinoma^20,21^, MCF10A is the benign, non-tumorigenic cell type(blue). In contrast, MDA-MB-231 and BT549 are the claudin-low, highly metastatic subtypes(red). MCF7 (luminal type A, light green), BT474 (luminal type B, green), SKBR3 (HER2-enriched, yellow) and MDA-MB-468 (basal type, orange) are the other four breast cancer subtypes with no to moderate *in vitro* metastatic potential^22^. The detailed clinicopathological features of the selected breast cell lines are listed in SI Table S1. From our TReD assay, we could clearly see that MDA-MB-231 and BT549 cells hadthe highest EGFR diffusivities (*D*) and the largest linear confinement sizes (*L*) (Fig. 1E,F). In particular, the EGFR diffusivity of MDA-MB-231 cell (0.0112 ± 0.0009 µm^2^/s, n = 800; the statistical estimator represents sample mean ± standard error of the mean) was 38% and 37% higher than those of MCF10A (0.0081 ± 0.0004 µm^2^/s, n = 2,598) and MCF7 (0.0082 ± 0.0004 µm^2^/s, n = 2,686) cells, respectively (Fig. 1E). Although not as differentiable as diffusivity, *L* of MDA-MB-231 cell (99.3 ± 4.9 nm, n = 800) was 23% and 11% larger than those of MCF10A (80.5 ± 2.6 nm, n = 2,598) and MCF7 (89.8 ± 2.9 nm, n = 2,686) cells, respectively (Fig. 1F). Around 15–20 trajectories were collected from each single cell, and at least 50 cells were tested in one cell line. Although the EGFR dynamics cannot differentiate the cancerous MCF7 cell from the non-tumorigenic MCF10A cell, a clear discrimination of the MDA-MB-231 and BT549 cells (highly invasive) from the MCF7, BT474, SKBR3, and MDA-MB-468 cells (non- to less invasive) is a remarkable evidence that the changes of TReD can be a signature of increased cancer invasiveness.

### Correlation between TReD and molecular signature

**Figure 2:**
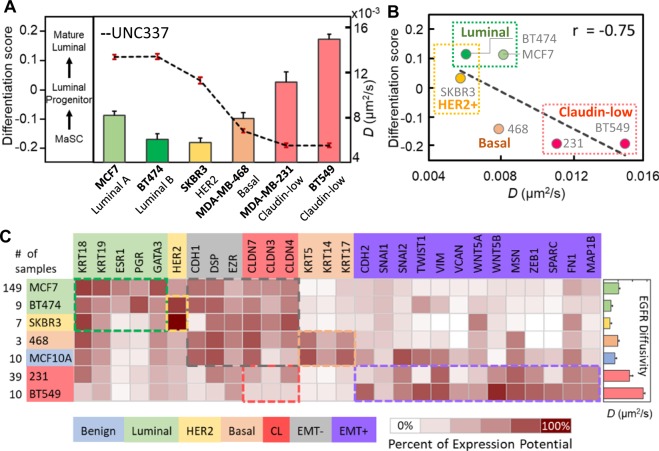
Correlation between TReD and molecular signature. (**A**) EGFR diffusivity versus luminal differentiation score. The luminal differentiation scores, as calculated on the basis of UNC337 gene expression database, represent the differentiation potentials of breast cells in a luminal epithelial differentiation lineage from mammary stem cells (MaSCs) to the luminal progenitor cells, and eventually to mature luminal cells. (**B**) The negative correlation between EGFR diffusivity and luminal differentiation score was seen, with the Pearson correlation coefficient r = −0.75. (**C**) Heat map generated from Genevestigator, showing the expression potential of the 28 genes related to EMT and the five breast cancer cell subtypes (luminal A, luminal B, HER2-rich, basal, and claudin-low). The highly invasive cancer cells exhibit features of mesenchymal cells. As higher EGFR diffusivities were observed in the highly invasive cells (MDA-MB-231 and BT549), we speculated that the EMT might alter the EGFR dynamics. Each cell subtype and the corresponding biomarkers have the same color code, and the colored boxes highlight the differences in molecular signatures among these seven cell lines. The *CLDN* genes are the biomarkers for claudin-low cells but also the EMT-downregulated genes.

By comparing our TReD results with the luminal differentiation scores (LD scores)^23^ of five distinct breast cancer subtypes (Fig. 2A), we found a clear negative correlation between the EGFR diffusivities and the LD scores (Pearson correlation r = −0.75, Fig. 2B). Calculated on the basis of UNC337 gene expression database (GSE18229 in GEO), the LD scores represent the potency of breast cells in a luminal epithelial differentiation lineage from mammary stem cells (MaSCs) to luminal progenitor cells, and eventually mature luminal cells^23,24^, where a lower or more negative LD score represents a higher differentiation potential of the cell. As the EGFR diffusivities are clearly (negatively) correlated with the LD scores, we can potentially use TReD to replace LD scores in quantifying the differentiation potency of cancer cells. To identify key differences in the regulatory networks involved in cancer metastasis among these seven breast cell lines, we scrutinized their gene expression profiles published in the Genevestigator microarray database^25^ (Fig. 2C and SI Tables S2 and S3). As expected, the gene expression patterns of the highly metastatic cells (claudin-low subtypes) showed signatures of EMT^26^ and features of cancer stem cells^27,28^. The highly invasive cell lines, MDA-MB-231 and BT549, expressed an increased levels of EMT-upregulated genes at a high level(the purple box in Fig. 2C), in contrast to the basal- and luminal-type of cells which expressed luminal cell-related and EMT-downregulated genes at a high level. We thus hypothesized that our TReD phenotyping results can be influenced by the expression of EMT-upregulated genes. In other words, EMT might change the dynamics of EGFRs.

### TReD assay monitors phenotypic transition of cells

**Figure 3:**
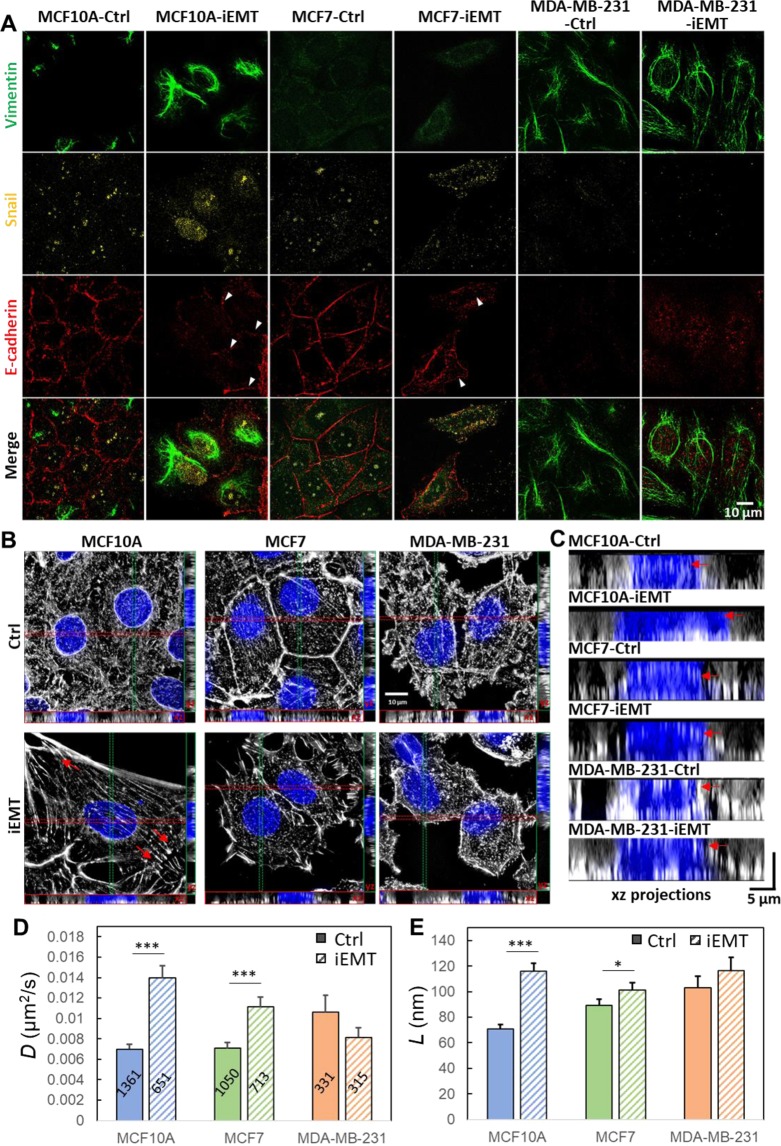
Characterization of EMT-induced cells and EGFR dynamics. (**A**) The phenotypes of the MCF10A, MCF7, and MDA-MB-231 cells treated with/without EMT induction medium (Ctrl and iEMT) were verified using immunocytochemistry with two mesenchymal biomarkers (snail and vimentin) and an epithelial biomarker (E-cadherin). As expected, after EMT induction, the mesenchymal biomarkers were up-regulated, while the epithelial biomarker was downregulated in both MCF10A and MCF7 cells (lower levels of E-cadherin pointed by the arrowheads). On the other hand, the expression levels of these three biomarkers in MDA-MB-231 cells remained the same before and after EMT induction, which reflects the fact that MDA-MB-231 is already a mesenchymal cell type. Scale bar represents 10 µm. (**B**) SR-SIM images of cortical actin. Maximum intensity projection on the xy plane, and orthogonal cross-sections (xz and yz) of MDA-MB-231, MCF7, MCF10A cells before and after EMT-induction. EMT induction clearly transformed the morphology of MCF10A and MCF7 cells (from the squamous shape to the spindle shape) and reorganized actin filaments (less cortical actins but more stress fibers). Having more stress fibers at the basal side of these chemically treated MCF10A and MCF7 cells indicated that these epithelial cells acquire fibroblast-like properties – a signature of EMT. The F-actin was labeled with Alexa Fluor 633 Phalloidin, and the nuclei were stained with Hoechst 33258. Scale bar represents 10 µm. (**C**) XZ projections of the SR-SIM images. The yellow dashed lines represent the apical borders of cells. **(D**,**E**) *D and L* were extracted from EGFR trajectories acquired from these cells. EMT-induction clearly facilitated the diffusion of EGFR in MCF10A and MCF7 cells (epithelial) but had no significant impact on MDA-MB-231 cell(mesenchymal). The number of trajectories analyzed is labeled on each bar in (D). Statistical comparison was performed using unpaired t-test, where the asterisk represents statistical significance ***p < 0.001, **p < 0.01, *p < 0.05. The error bar represents the standard error of the mean.

To test the hypothesis that EMT can regulate the organization of cortical actin network and change the EGFR dynamics, we chemically induced EMT in three breast cell lines (non-tumorigenic MCF10A, non-invasive MCF7, and highly invasive MDA-MB-231) using the commercial EMT induction medium (StemXVivo EMT Inducing Media Supplement) (Fig. 3A–C) and measured TReD before and after the induction (Fig. 3D,E). The effectiveness of EMT induction was verified by an EMT immunochemistry kit (SC026, R&D systems) which identifies snail, vimentin, and E-cadherin (Fig. 3A) and imaged with a Structured Illumination Super-Resolution Microscope (SR-SIM). In EMT-induced MCF10A and MCF7 cells, the decrease of E-cadherin (pointed by the arrowheads in Fig. 3A) resulted in reduced intercellular adhesion and increased cell motility^29^. In addition, upon EMT induction, cadherins could incidentally establish a link with the actomyosin cytoskeleton through α- and β- catenin, and a decrease of cadherin-containing cell-cell junctions could reduce cortical tension and increase actin turnover^30^. As a result, we observed clear morphological transformations in EMT-induced MCF10A and MCF7 from squamous cells to spindle-shaped cells which expressed less cortical actin but more stress fibers (Fig. 3B). In tumor progression, cells that undergo EMT reorganize their cortical actin cytoskeleton into stress fibers or membrane projections, which enables dynamic cell elongation and directional motility^31–33^. The EMT-induced MCF10A clearly exhibited actin-rich membrane projections that facilitate cell movement and act as sensory extensions of the cytoskeleton (pointed by the arrows in Fig. 3B). These projections included lamellipodia (sheet-like membrane protrusions) and filopodia (spike-like extensions) at the edge of lamellipodia^34^. In particular, the actin-rich invadopodia could exert a proteolytic function in extracellular matrix degradation, facilitating cell invasion^34^. As MCF10A and MCF7 cells became similar to MDA-MB-231 cell after EMT induction, we expected to see higher EGFR diffusivities and larger linear confinement sizes. Indeed, EMT-induced MCF10A and MCF7 cells showed a marked increase in EGFR diffusivities (0.0139 ± 0.0012 µm^2^/s, n = 651; 0.0111 ± 0.0010 µm^2^/s, n = 713), which were substantially higher (99% and 56%) than those of the untreated controls (0.0070 ± 0.0005 µm^2^/s, n = 1361; 0.0071 ± 0.0006 µm^2^/s, n = 1,050) (Fig. 3D). In contrast, the MDA-MB-231 cell showed a decrease in EGFR diffusivity (0.0106 ± 0.0016, n = 331 to 0.0081 ± 0.0009 µm^2^/s, n = 315) after EMT induction (by 23%, but without a statistical significance). On the other hand, the membrane compartment size (*L*) increased in all three cells after EMT induction, although the changes were less statistically significant for MCF7 and MDA-MB-231 cells (Fig. 3E). We emphasize that the highly invasive cells possess the features of mesenchymal cells, as MDA-MB-231 and BT549 cells express a high level of EMT-upregulated genes (Fig. 2C). The results of our EMT induction studies (Fig. 3) resonated well with the cell line studies (Fig. 1). TReD assay can, therefore, probe the transition of cells from the pre-malignant state to the highly invasive state.

### TReD assay provides detailed information about the receptors

**Figure 4:**
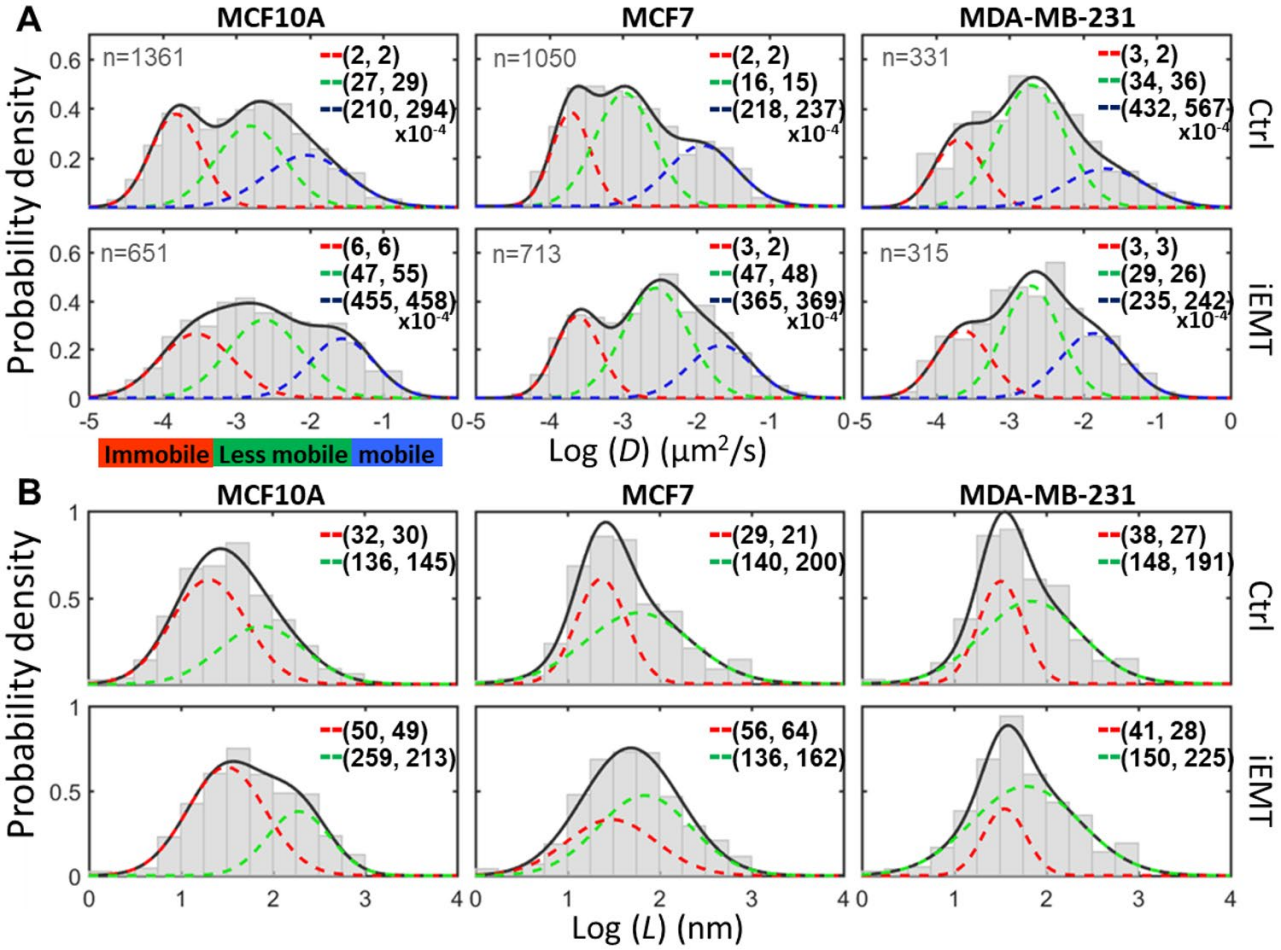
EMT changes the diffusivity of EGFR and the size of microdomains. Histograms of log *D* (**A**) and log *L* (**B**) extracted from EGFR trajectories. The histograms reveal the heterogeneity of EGFR dynamics and the change of EGFR dynamics upon EMT. EMT induction clearly reshaped the population distributions among the three different diffusive states (immobile, less mobile, and mobile). In MCF10A and MCF7 cells, both EGFR diffusivities and the compartment sizes increased after EMT induction. The values shown in the histograms represent the arithmetic moments of *D* or *L* ((arithmetic means ($\bar{X}$), arithmetic strandard deviations(${\sigma }_{\bar{x}}$))) derived from Gaussian mixture model fitting of log *D* and log *L* (with 3 or 2 sub-populations). The three subpopulations are defined as immobilization (red), less mobile (green), and mobile (blue). The number of trajectories analyzed from each group is shown in (**A**).

The histograms of log *D* and log *L* revealed the detailed information on the heterogeneity of EGFR dynamics and the change of EGFR dynamics upon EMT (Fig. 4). The log *D* plot clearly showed three diffusive states (immobile, less mobile, and mobile states) for the three cell lines (Fig. 4A), and EMT induction changed the profiles of three-component Gaussian fits. It is well known that the dynamic properties of single member receptors often vary in a wide range of spatial scales (from tens to hundreds of nanometers) and temporal scales (from a few milliseconds to seconds)^35,36^. This complex behavior is caused by a combination of factors^37^, including molecular crowding^38^, molecular interactions^16,39^, membrane topology^40^, and interactions with nanostructures within the membrane domains (e.g., caveolae^41,42^, clathrin-coated pits^43^, lipid rafts^44^, and cytoskeleton corrals^35,45–47^). The MCF10A and MCF7 cells had their mobile-state diffusivities substantially increased (blue dash lines, 117% & 67%, respectively), but the mobile-state diffusivity of MDA-MB-231 cell decreased after EMT induction (45%). On the other hand, the log *L* histograms of the benign (MCF10A) and the non-invasive (MCF7) cells, once fitted with a two-component Gaussian model, showed a substantial increase for the smaller *L* component after EMT induction (red dash lines, by 56% & 93%, Fig. 4B). However, such a significant increase in the linear confinement size was not seen in the highly invasive cell (MDA-MB-231). We noted that the average track duration for a single EGFR complex was about 30 seconds (all tracks shorter than 15 s were discarded) and within 30 seconds the EGFR complex could have switched among different diffusive states multiple times (especially for MCF10A and MCF7 cells). We have previously observed an interchange of four diffusive states of EGFR (Brownian diffusion, confined diffusion, directed diffusion, and immobilization) in A431 cells (upon EGF stimulation; here all of the experiments were done without EGF stimulation) using a 3D-SPT technique^19^. We emphasize that other physical science approaches provide no detailed description of the receptor motion and the microenvironment where the receptors are contained, and shed no light on how the receptor behavior can change during the phenotypic transition. Based on the evidence that both the mobile-state EGFR diffusivities and the nanoscopic confinement sizes increased after EMT induction, we concluded that the cortical actin network was reorganized during EMT.

### Depolymerization of F-actin increases EGFR diffusivity and enlarges confinement size in benign cells

**Figure 5:**
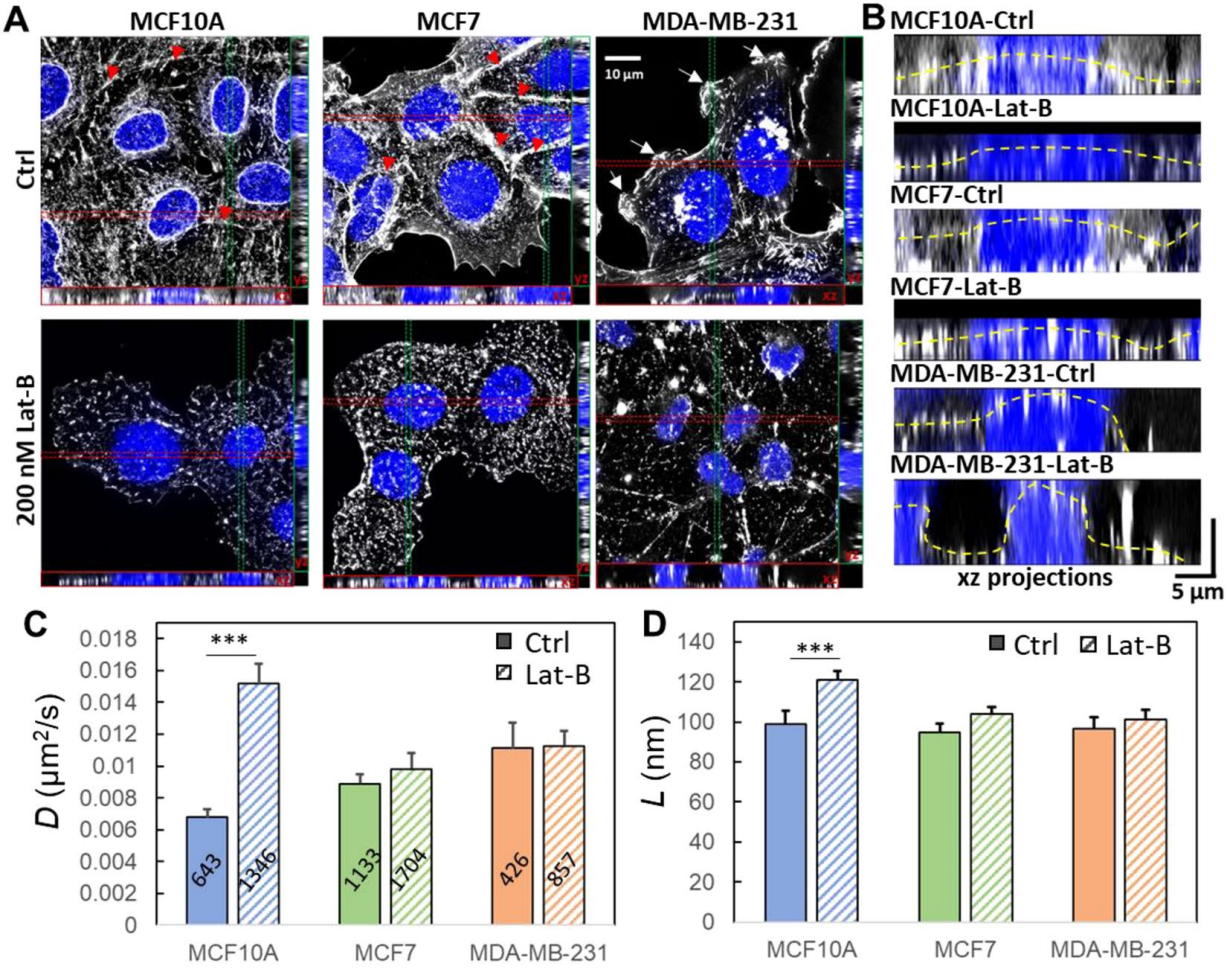
Depolymerization of F-actin increases EGFR diffusivity and enlarges confinement size. (**A**) Maximum intensity projection on the xy plane, and orthogonal cross-sections (xz and yz) of MDA-MB-231, MCF7, and MCF10A cells before and after Lat-B treatments. Lat-B indeed inhibited the polymerization of F-actin. The red arrowheads indicate the peri-junctional actin bands, while the white arrows pinpoint the filopodia-like structures. (**B**) The xz projections clearly show the dissociation of cortical actin from the apical surface of the plasma membrane after the treatment. The yellow dashed lines represent the apical borders of cells. (**C**) Diffusivities of EGFR extracted from trajectories. (**D**) Linear dimension of the confinement extracted from trajectories. The number of trajectories analyzed is labeled on each bar in (**C**). Statistical comparison was performed using unpaired t-test, where the asterisk represents statistical significance: ***p < 0.001, **p < 0.01, *p < 0.05. The error bar represents the standard error of the mean.

To investigate how the cortical actin structure might influence EGFR dynamics, we treated the three cell lines with Latrunculin B (Lat-B) that depolymerizes F-actin^48^. Before treatment, MCF10A and MCF7 cells were featured by abundant peri-junctional actin bands (pointed by arrowheads in Fig. 5A) and denser cortical actin networks in their apical PM (Fig. 5B). In contrast, MDA-MB-231 cell exhibited many filopodia-like structures (pointed by arrows in Fig. 5A). Upon Lat-B treatment, substantial disruption of stress fibers, reduction of cortical actin, retraction of filopodia, and decrease in projected cell area were observed in all three cell lines (Fig. 5A). The XZ projections clearly showed the dissociation of cortical actin from the apical surface of the PM after treatment (Fig. 5B). This reorganization of cortical actin meshwork was clearly responsible for the increased diffusivity of EGFR(Fig. 5C) and the enlarged confinement size (Fig. 5D) in the Lat-B-treated MCF10A cells. While Lat-B also depolymerized the F-actin in MCF7 cells, especially at the cell-cell contact (Fig. 5A), the change in EGFR diffusivity was marginal (Fig. 5C). Interestingly, the Lat-B treatment did not affect the EGFR diffusivity in MDA-MB-231 cell. The log *D* and log *L* histograms of the Lat-B treated cells are shown in SI Fig. S2.

### EMT-induced actin reorganization suppresses EGF-dependent tyrosine phosphorylation in MCF10A cell

**Figure 6:**
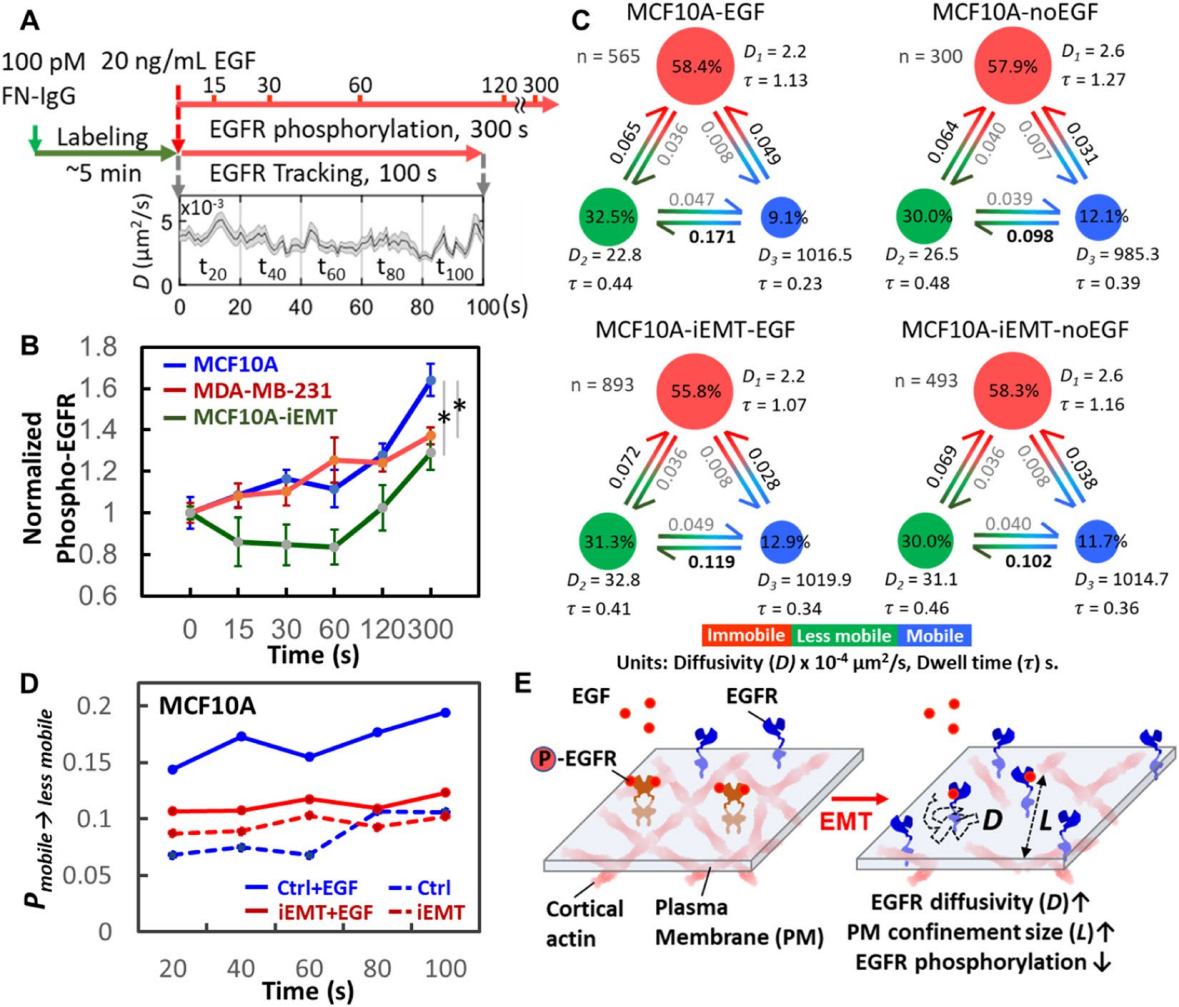
Relaxation of EGFR confinement reduced EGF-induced EGFR phosphorylation. (**A**) Procedure to evaluate the connection between EGFR dynamics and EGFR phosphorylation. For EGFR phosphorylation, cells were stimulated with or without EGF (t = 0 s), and the phosphorylation was measured at different time points. EGFR tracking was immediately initiated after the addition of cell culture medium containing EGF. The plot shows the time trace of EGFR diffusivity. (**B**) EGF-stimulated EGFR phosphorylation in MCF10A, EMT-induced MCF10A, and MDA-MB-231 cells. Three trials were conducted. In each trial, 4 data points were collected at one time point. Statistical comparison was performed using unpaired t-test, where the asterisk represents statistical significance: *p < 0.05. The error bar represents the standard error of the mean. (**C**) vbSPT analysis revealed the dynamics of EGFRs in MCF10A cell under the perturbation of EMT-induction or EGF-stimulation. The three diffusive states are defined as immobilization (red), less mobile (green), and mobile (blue).Transition probabilities, state occupancies, average dwell time (τ), and number of trajectories analyzed are shown in each subplot. (**D**) The transition probability from the mobile to the less mobile (*P*_*Mobile→Less mobile*_). Trajectories were truncated into five time windows and analyzed by vbSPT to reveal the changes of *P*_*Mobile→Less mobile*_ every 20 s. (**E**) Schematic shows our hypothesis of the effect of EMT on the dynamics of EGFRs and EGFR phosphorylation.

Many studies have demonstrated that spatiotemporal confinement of the membrane receptors facilitates oligomerization of the membrane receptors and their associated molecules^16,49^ and further enhances signaling in the compartment^3,35,50,51^. Therefore, we speculated whether the EMT-induced actin reorganization would impact EGFR phosphorylation. Here we focused on MCF10A cell and estimated the level of EGFR phosphorylation using a commercial kit (Phospho-EGFR Cellular Assay, Cat. # 64EG1PEG, cisbio) at 6 different time points after EGF stimulation (0 s, 15 s, 30 s, 60 s, 120 s, and 300 s). Starting from time zero (EGF stimulation), we also tracked EGFR dynamics for 100 seconds (Fig. 6A). We found that the EGFR phosphorylation levels in the EMT-induced MCF10A cell (with less EGFR confinement) were indeed lower than those in the untreated cells over the course of 5 minutes (Fig. 6B), suggesting that EGFRs might have become desensitized. To reveal the connection between EGFR phosphorylation and EGFR dynamics, we use an analytical tool, variational Bayes single-particle tracking (vbSPT)^52^, to characterize the transitions between different EGFR diffusive states (Fig. 6C). vbSPT identified three diffusive states (immobile, less mobile, and mobile) and provided state occupancies and transition probabilities among states. We found EMT induction greatly suppressed the transition probability from the mobile to the less mobile state (*P*_*Mobile→Less mobile*_) after EGF stimulation (Fig. 6C and 6D), which agreed well with our previous observation that EMT induction relaxed the physical confinement of EGFR, leading to a higher overall EGFR diffusivity. Together, we concluded that EMT-induced actin reorganization enlarges the meshwork confinements, thus reducing EGF-dependent tyrosine phosphorylation (Fig. 6E). The increase in *D* and *L* and the decrease in *P*_*Mobile→Less mobile*_ also suggest less EGFR dimerization and less association between EGFR and actin proteins after EMT.

## Discussion

To the best of our knowledge, transmembrane receptor dynamics (TReD) have never been used to distinguish cancer cells with distinct metastatic potentials. We followed the structure-property-function-disease paradigm proposed by Suresh^53^ and established connections among EGFR dynamics, cancer metastasis, EMT, cortical actin structures, and EGFR phosphorylation. Here we demonstrate that EGFR dynamics can serve as a physical biomarker to distinguish highly-invasive breast cancer cells (MDA-MB-231 and BT549) from other subtypes (Fig. 1), to monitor phenotypic transition of cells (e.g., EMT in MCCF10A, Fig. 3D), and to probe the changes in the cellular microenvironment (reorganization of cortical actin meshwork in Fig. 3E). As mechanical force, spatial organization of surface receptors, and receptor-mediated signal transduction (which leads to increased metastatic potential) are all coupled^3^, chemical states of the cell can influence the physical states of the cell and vice versa^54,55^, which lay the foundation of our TReD assay.

There are many benefits of using TReD to monitor the alteration of cell propeties, such as cytoskeletal changes and mechanical propety changes. While the cytoskeletal changes can be directly visualized by methods such as structured illumination microscopy, tedious cell fixation and staining with external fluorophores (e.g., Alexa-633 Phalloidin) are often needed. TReD, in contrast, provides a quick and simple way to evaluate cytoskeletal changes in live cells. While the mechanical properties of cancer cells can be measured by AFM^5^ or micropipette aspiration^10^, the requirement to physically touch the cells makes these methods incompatible with typical microfluidic devices. On the contrary, TReD is based on single-particle tracking that can be easily performed in many commercially available microfluidic cell-sorting devices (e.g., *Parsortix* from Angle PLC for CTC isolation^56^, SI Fig. S3). In this report, we focus on two physical parameters (*D* and *L*) that most successfully distinguish the malignant subtypes (MDA-MB-231 and BT549) from the other subtypes, while the discrimination among non- to less-invasive subtypes is currently marginal. However, we emphasize that much more information about the receptors, such as endocytosis process, active transport, and dimerization kinetics, can be acquired using more sophisticated 3D-SPT techniques^57–61^ or two-color colocalization techniques^16,62,47,58^. Although we have previously advanced 3D-SPT techniques^63^ and reported a wealth of information about EGFR trafficking, from PM to cytosol, in skin cancer A431 cells^19^, 3D-SPT is currently limited by its low throughput (tracking one receptor complex at a time). Bypassing this low throughput issue, here we demonstrate the TReD assay based on 2D-SPT. Our next goal is to extend TReD assay from 2D to 3D and incorporate more dynamical parameters that can further differentiate the four non- to less-invasive subtypes (MCF7, BT474, SKBR3, and MDA-MB-468). In addition, we are also aware that cross-linking effect from IgG antibodies or multivalent nanoparticles could have impacted EGFR dynamics. Therefore, using advanced monovalent nanoparticles^64,65^ for tracking of single receptors is excpeted to improve the performance of TReD assay.

We notice that the HER2-positive breast cancer cells, BT474 and SKBR3, had the lowest EGFR diffusivities among all seven cell lines tested in Fig. 1E. This result resonates with Agazie’s report that overexpression of *HER2* stabilizes HER2 homodimers and HER2-EGFR heterodimers^66^. Following ligand binding, the EGFR family receptors (EGFR/HER1/ErbB-1, HER2/ErbB-2/neu, HER3/ErB-3, and HER4/ErbB-4) interact to form an array of homo- and heterodimers^67^. In particular, in cells expressing both EGFR and HER2, ligand stimulation induces both EGFR-EGFR homodimerization and EGFR-HER2 heterodimerization^68^. The activated dimers further recruit binding proteins to enhance signaling^2,69^, thus resulting in a bigger EGFR complex with reduced diffusivity^51^. To assess the correlation between the expression levels of EGFR family receptors and EGFR dynamics, we analyzed the gene expression data of the four EGFR family receptors (*EGFR*, *HER2*, *HER3*, and *HER4*) and compared that to the EGFR diffusivity (Fig. S4). Interestingly, we found no strong correlation between EGFR diffusivity and EGFR expression level (r = −0.05). In contrast, the *HER2* and *HER3* gene expression levels are clearly negatively correlated with EGFR diffusivity (r = −0.6 and r = −0.67, respectively). We emphasize that, although interesting, this result needs to be reconfirmed in isogenic cell models where the expression levels of each EGFR family receptor are well-controlled and characterized, such as conducting EGFR tracking in a genetically modified MCF10A cell with a well-regulated HER2 expression level.

Many research groups have demonstrated that the cortical actin compartmentalizes the PM into microdomains (~40–300 nm in diameter^70^) which temporally confine transmembrane receptors from a few milliseconds to hundreds of milliseconds. This spatiotemporal confinement facilitates oligomerization of the receptors and their associated molecules^16,49^ and enhances signaling in the microdomains^3,35,50,51^. We have shown that EMT relaxs the EGFR confinement (Fig. 2), which decreases the oligomerization of receptors and results in a lower level of tyrosine phosphorylation (Fig. 6). However, we emphasize that spatial confinement of transmembrane receptors does not always enhance signaling. For instance, cortical actin can also act as barriers to prevent receptor oligomerization, restricting the antigen-stimulated signaling in immune cells^71,72^. In other words, cortical actin could have two distinct effects in regulating membrane receptor signaling. On one hand, it may facilitate the clustering of receptors and downstream effectors to enhance cell signaling. On the other hand, it can separate receptors from each other and prevent the initiation of cell signaling. In addition to the confinement size, what also plays a critical role here is the number of receptors in each confinement (i.e., the expression level of receptors). As mentioned above, future TReD assay will collectively characterize confinement size, receptor diffusivity, and receptor density on the PM in differentiating cancer subtypes. Other than the physical constraints from the intracellular domain, the extracellular matrix can also modulate the dynamics of transmembrane proteins. Grinstein's group has recently demonstrated that extracellular matrix regulates the dynamics of transmembrane proteins such as CD44 and Fcγ receptor^73^. We are adapting the TReD assay to study *in vivo* models through intravital microscopy, which will shed light on how extracellular matrix, interstitial fluid, and cell-cell interactions affect receptor dynamics.

In conclusion, we present a new type of biophysical phenotyping assay - termed TReD which is capable of differentiating the highly-invasive cancer cell lines (MDA-MB-231 and BT549) from the non- to less-invasive cancer cell lines. Through the combined interpretation of TReD and structured illumination microscopy images, we show that EMT reorganizes cortical actin and modulates the dynamics of EGFRs. Finally, we demonstrate that the EMT relaxes the confinement of EGFRs and attenuates the EGF-induced tyrosine phosphorylation of EGFR. However, the detailed mechanism of how the cortical cytoskeleton serves as a guardian to upregulate or downregulate cell signaling remains to be answered. We envision that a collective, systematic investigation on (i) the compartmentalization of PM in single cells and (ii) the density and dynamics of receptors in single compartments across a variety of cells wouldelucidate the role of the cortical cytoskeleton in transmembrane signaling.

## Methods

### Cell culture

Used as the model systems, BT474, SKBR3, MDA-MB-468, MDA-MB-231, and BT549 cells were grown in DMEM/F12 medium (11320082, Thermo Fisher Scientific) supplemented with 10% fetal bovine serum (16140071, Thermo Fisher Scientific) and 50 U/mL penicillin-streptomycin (15070063, Thermo Fisher Scientific). MCF7 was grown in MEM (11095-080, Thermo Fisher Scientific) supplemented with 10% fetal bovine serum and 50 U/mL penicillin-streptomycin. The benign breast epithelial cell line, MCF10A, was maintained as described^69^. Please see SI Method S1 for a detailed description of cell culture.

### EMT induction and depolymerization of actin filaments

To induce EMT, MCF10A, MCF7, and MDA-MB-231 cells were grown in their original media that were additionally supplemented with the EMT induction supplement (StemXVivo EMT Inducing Media Supplement, Cat. No. CCM017, R&D systems) for five days^74^. The supplement includes anti-human E-cadherin, anti-human sFRP-1, anti-human Dkk-1, recombinant human Wnt-5a, and recombinant human TGF-β. The cells were characterized by an EMT immunochemistry kit (SC026, R&D systems) which contains three types of IgGs targeting snail, E-cadherin, and vimentin, respectively. Latrunculin B (Lat-B, ab144291, Abcam) was used to depolymerize actin filaments^48^. For Lat-B-treated cells, cells were incubated with 200 nM Lat-B supplemented in serum-free DMEM/F12 or MEM medium for 10 min.

### Fluorescent probe labeling to EGFR

Anti-EGFR IgG antibody-conjugated fluorescent nanoparticles (FN-IgG) were used to label EGFR for tracking. The FN-IgG probe was prepared as described by us previously^19^. Full details of fluorescently labeling EGFR can be found in SI Method S2.

### Single-particle tracking and trajectory analysis

Wide-field imaging for SPT is performed using an Olympus IX71 inverted microscope equipped with a 60x 1.2 N.A. water objective (UPLSAPO 60XW, Olympus). All imaging was conducted at 37 °C using a temperature-controlled stage (Stable Z System, Bioptechs). Wide-field excitation was provided by a metal halide lamp with a 545/25 nm BP excitation filter. Emission was collected by a Scientific CMOS camera (ORCA-Flash4.0) through 565 nm dichroic and 605/70 BP. The pixel size is equivalent to 107 nm. Images of FN-IgG tagged EGFRs (FN-IgG-EGFRs) were acquired at 20 frames per second for a total of 1,200 frames (TReD assay) or 2,000 frames (EGF-stimulation). The analysis of the acquired image series was performed as described previously^47,75^ to obtain trajectories (SI Method S3). The SPT software was a gift from Prof. Keith Lidke at the University of New Mexico. The trajectories were analyzed using MSD analysis to extract the EGFR diffusivity (*D*) and the linear size the confinement (*L*). To reveal the multiple transitions of EGFRs among the distinct diffusive states, trajectories were further analyzed with variational Bayes SPT (vbSPT)^52^ to identify the number of diffusive states and the state transition probabilities. Please see supplementary information for detailed descriptions of single-particle tracking (SI Method S4) and trajectory analysis (SI Method S5).

### Gene expression analysis of breast cancer cell microarray dataset

The luminal differentiation predictor was developed by Perou group^24^ and trained with the database GSE16997 which is contributed by Partanen group^23^. Then this predictor was applied to the UNC337 database (GSE18229 in GEO) to calculate the luminal differentiation scores (LD scores) of 39 tumor samples (*n* = 337). The calculation of LD score is described in SI Method S6. A search engine for gene expression, Genevestigator^25^, was used to identify the gene expression patterns of these selected cell lines in a large scale, and 26 datasets and 28 genetic markers were included in the analysis. For data selection, we selected the gene expression data of these seven cell lines without any perturbations, such as drug treatments, gene knock-in/knock-down. Twelve classical markers used to characterize breast tumors were included to reveal the gene signatures across the intrinsic breast cancer subtypes. In addition, 19 EMT up- or down-regulated genes were selected to reveal the EMT features. Please see SI for the list of the sources of the gene expression data (SI Table S3) and the list of the selected genes (SI Table S4).

### Immunofluorescence and structured illumination microscopy

Cells were grown on an 8-well chambered coverglass (154534, Thermo Scientific), fixed with 4% formaldehyde (F8775, Sigma-Aldrich), and permeabilized with 0.1% Triton X-100/PBS (Triton X-100, T8787, Sigma-Aldrich) prior blocking with 1% BSA in PBS. Then, the samples were incubated with antibodies overnight at 4 °C. To assess EMT status, cells were characterized by an EMT immunochemistry kit (SC026, R&D systems). The Alexa Fluor 633 Phalloidin (A22284, ThermoFisher Scientific) was used to stain actin filaments (F-actin). The fluorescence imaging was taken by Elyra S.1 Structured Illumination Super-Resolution Microscope (SR-SIM) with a 63x 1.2 N.A. water objective. Please see SI Method S7 for detailed information.

### Statistical analysis

The unpaired two-sample t-tests were used to determine whether two sets of diffusivities (*D)* or linear sizes of compartments (*L)* were significantly different from each other of two groups. The F-test was used to check whether the two samples have the same variance, then according to the result of F-test, unpaired two-sample t-test with either equal variance or unequal variance was applied to test the null hypothesis: the means (*D* or *L*) of two populations are equal. Rather than a normal distribution, lognormal distribution was often used to describe the broad distribution of particle-trajectory-derived diffusivity^76,77^. The central limit theorem can be applied in the t-tests because of the great numbers of trajectories we collected in our study (at least 300 trajectories), and the t-test is valid even when *D* and *L* follow a lognormal distribution. The t-test is based on the two groups means $\overline{{X}_{1}}$ and $\overline{{X}_{2}}$. Because of the central limit theorem, the distribution of $\overline{{X}_{1}}$ and $\overline{{X}_{2}}$, in repeated sampling, converges to a normal distribution, irrespective of the distribution of *X* in the population^78^. Thus t-test is able to be used to test *D* and *L* among cell lines or cell lines with different treatments. In Figs 1–6, the error bar represents standard error of the mean $(\frac{Standard\,deviaiton}{\sqrt{size\,of\,the\,sample\,}})$.

Many groups have shown that the transmembrane receptors exhibit complex dynamics^19,79,^^80^.It is therefore common to fit the diffusivity histograms of the receptors with lognormal distribution and Gaussian mixture model^81^. Here we used the MATLAB built-in function, fitgmdist, to fit the histograms and evaluated the goodness of fit by the Akaike information criterion (AIC)^82,83^. The best-fitted number of components were decided by the fitting with the smallest AIC. From our previous study^19^, three major populations were often seen in the EGFR diffusivity distribution. We evaluated the goodness of fit in one to three components, and presented the fitting results with the lowest AIC. The fitting means and standard deviations of log *D* were used to derive the arithmetic means and arithmetic standard deviations of *D*. Please see SI Method S8 for the derivation of these two arithmetic moments.

### Homogeneous time-resolved fluorescence (HTRF) analysis

Quantification of phosphorylated EGFR levels was performed using the HTRF-Cellular kits: Phospho-EGFR (Tyr1068) Cellular Assay Kit (64EG1PEG, cisbio) and Total EGFR Cellular Assay Kit (64NG1PEG, cisbio). The specific EGFR phosphorylation on Tyr1068 and the total EGFR were measured after EGF-stimulation (20 ng/mL EGF (recombinant human epidermal growth factor, PHG0311L, Thermo Fisher Scientific) in serum-free DMEM/F12 medium). We followed the two-plate assay protocol provided by the vendor (SI Method S9 for a detailed description).

## Supplementary information

Supplementary information
