# Supplementary material for: Assessing metastatic potential of breast cancer cells based on EGFR dynamics
Source: Sci Rep. 2019 Mar 4;9:3395. doi: 10.1038/s41598-018-37625-0 (PMC6399327; doi:10.1038/s41598-018-37625-0)
Supplement: Supplementary file 1 — Supplementary information [file 41598_2018_37625_MOESM1_ESM.docx]

**Supplementary Information**

Assessing metastatic potential of breast cancer cells based on EGFR dynamics

Yen-Liang Liu^1^, Chao-Kai Chou^2^, Mirae Kim^1^, Rohan Vasisht^1^, Yu-An Kuo^1^, Phyllis Ang^3^, Cong Liu^1^, Evan P. Perillo^1^, Yu-An Chen^1^, Katherine Blocher^1^, Hannah Horng^4^, Yuan-I Chen^1^, Duc Trung Nguyen^1^, Thomas E. Yankeelov^1,5,6,7,8^, Mien-Chie Hung^2,9^, Andrew K. Dunn^1^, & Hsin-Chih Yeh*^,1^

^1^Department of Biomedical Engineering, The University of Texas at Austin, Austin, TX, USA

^2^Department of Molecular and Cellular Oncology, The University of Texas MD Anderson Cancer Center, Houston, TX, USA

^3^Department of Electrical and Computer Engineering, The University of Texas at Austin, Austin, TX, USA

^4^Department of Bioengineering, The University of Maryland, College Park, MD, USA

^5^Institute for Computational Engineering and Sciences, The University of Texas, Austin, TX, USA

^6^Department of Diagnostic Medicine, Dell Medical School, The University of Texas at Austin, Austin, TX, USA

^7^Department of Oncology, Dell Medical School, The University of Texas at Austin, Austin, TX, USA

^8^Livestrong Cancer Institutes, The University of Texas at Austin, Austin, Texas, USA

^9^Center for Molecular Medicine and Graduate Institute of Cancer Biology, China Medical University, Taichung, Taiwan

^10^Texas Materials Institute, The University of Texas at Austin, Austin, TX, USA

Content

[**Method S1 | Cell culture** 2](#_Toc527420812)

[**Method S2 | Fluorescent probe labeling to EGFR** 2](#_Toc527420813)

[**Method S3 | Data processing** 2](#_Toc527420814)

[**Method S4 | Single Particle Tracking** 3](#_Toc527420815)

[**Method S5 | Extracting dynamic parameters from MSD** 3](#_Toc527420816)

[**Method S6 | Calculation of luminal differentiation score** 4](#_Toc527420817)

[**Method S7 | Immunofluorescence and structured illumination microscopy** 4](#_Toc527420818)

[**Method S8 | Derivation of arithmetic moments from normally distributed log *D* and log *L*** 5](#_Toc527420819)

[**Method S9 | Homogenous time-resolved fluorescence (HTRF)** 5](#_Toc527420820)

[**Figure S1 | Assays for biophysical characterization of cells** 7](#_Toc527420821)

[**Figure S2 | LatB changes the diffusivity of EGFR and the size of microdomains.** 8](#_Toc527420822)

[**Figure S3 | Cassette compatibility of TReD assay** 9](#_Toc527420823)

[**Figure S4 | Correlations between EGFR diffusivity and expression levels of EGFR family** 10](#_Toc527420824)

[**Table S1 | Molecular classification, clinical and pathological features, and sources of cell lines** 11](#_Toc527420825)

[**Table S2 | Source of gene expression data (mRNA microarray)** 12](#_Toc527420826)

[**Table S3 | Selected genes for gene expression analysis** 13](#_Toc527420827)

**[Supplementary References](#_Toc527420828)** [14](#_Toc527420828)

## **Method S1 | Cell culture**

BT474, SKBR3, MDA-MB-468, MDA-MB-231, BT549 were grown in DMEM/F12 medium (11320082, Thermo Fisher Scientific) supplemented with 10% fetal bovine serum (16140071, Thermo Fisher Scientific) and 50 U/mL penicillin-streptomycin (15070063, Thermo Fisher Scientific). MCF7 was grown in MEM (11095-080, Thermo Fisher Scientific) supplemented with 10% fetal bovine serum and 50 U/mL penicillin-streptomycin. The benign breast epithelial cell line, MCF10A, was maintained as described [^1^](#_ENREF_1). The recipe of MCF10A medium is DMEM/F12 medium supplemented with 5% horse serum (16050122, Thermo Fisher Scientific), 20 ng/mL epidermal growth factor (AF-100-15, Peprotech), 0.5 μg/mL hydrocortisone (H0888, Sigma-Aldrich), 100 ng/mL cholera toxin (C8052, Sigma-Aldrich), 10 μg/mL insulin (I9278, Sigma-Aldrich), and 50 U/mL penicillin-streptomycin. These cells were kept in a humidified atmosphere with 5% CO2 in air at 37ºC. Single suspensions were prepared by mild enzymatic dissociation using a 0.25% trypsin/EDTA solution (25200-056, Thermo Fisher Scientific). For immunofluorescence and SPT experiments, cells were grown on an optical imaging 8-well chambered coverglass (154534, Thermo Scientific).

## **Method S2 | Fluorescent probe labeling to EGFR**

Anti-EGFR IgG antibody-conjugated fluorescent nanoparticles (FN-IgG) were used to label EGFR for tracking. Biotinylated monoclonal anti-EGFR antibodies (Clone Ab-3, MS-311-B, Thermo Fisher Scientific) were mixed at 1:1 ratio with ⌀40 nm NeutrAvidin-labeled red fluorescent nanoparticles (F8770, Thermo Fisher Scientific) in 1.5% bovine serum albumin (BSA, S7806, Sigma-Aldrich) in PBS solution. The Ab-3 does not interfere with the binding of EGF to EGFR. The FN-IgG (30 nM for the stock solution) can be stored at 4ºC for up to 1 week. The number of antibodies per nanoparticle should follow a Poisson distribution[^2^](#_ENREF_2). For EGFR tracking, cells were seeded onto optical imaging 8-well chambered coverglass (154534, Thermo Fisher Scientific) with a cell density of 1×10^5^ cells per well and allowed to grow to 70-80% cell confluency. Before tracking experiments, cells were stained with Hoechst 33258 (H3569, Thermo Fisher Scientific, 1:1000 dilution in cell culture medium) for 10 minutes at 37ºC. Then, the staining buffer was replaced with the EGFR-labeling solution (FN-IgG at 100 pM) diluted from the stock solution (30 nM). The reaction was incubated for 10 minutes at 37ºC. The EGFR-labeling solution was removed, and the samples were washed twice using PBS to remove the unbound fluorescent nanoparticles. The volumes of all solutions and washing buffers used in staining were 200 µl per well. Upon completion of nucleus staining and EGFR labeling, the chambered coverglass was immediately put on the microscope for tracking experiments. All imaging was conducted at 37 °C using a temperature-controlled stage (Stable Z System, Bioptechs).

## **Method S3 | Data processing**

All data analysis and image processing were performed within the MATLAB (The Mathworks Inc.) environment, including the DIPImage^[3](#_ENREF_3)^ image processing library. The images were taken with HCImage (a Hamamatsu's image acquisition and analysis software), and the single particle trajectories were reconstructed by using a single-particle tracking software developed by Lidke’s group[^4^](#_ENREF_4). Later, the trajectories were processed into mean-squared displacement curves where the diffusion coefficient (*D*) and the linear dimension of the compartment (*L*) were extracted by curve fitting (**Method S5**, also see our previous publication[^5^](#_ENREF_5) for a detailed description).

## **Method S4 | Single Particle Tracking**

Single particle trajectories were determined from the raw data sets using a three-step process: (i) Identifying contiguous regions of pixels; (ii) Gaussian fitting; (iii) building trajectories from coordinates. This approach is similar to those described previously[^4^](#_ENREF_4)^,^ [^6^](#_ENREF_6)^,^ [^7^](#_ENREF_7).

**1. Identifying continugous regions of pixels.** A series of 2D images in time trace was processed independently to find FN-IgG-EGFR coordinates. The contiguous regions of pixels, which represent the images of fluorescent particles, were identified on the basis of two criteria: (i) pixels had intensities greater than 3-fold the standard deviation of pixel intensities from areas defined as background (background offset algorithm[^3^](#_ENREF_3)) and (ii) pixels were above a threshold[^3^](#_ENREF_3). Then, a high pass filtering was applied to the image with a 2D Gaussian filter (σ = 5). The binary image of pixels passing both criteria was later processed by Gaussian fitting.

**2. Gaussian fitting.** To find out the center of the fluorescent particles, the center of mass of each contiguous region in the binary image was set as the starting point in a Gaussian fitting routine. The highest intensity pixel in a small region around the starting point (5 pixels square) was used as an updated starting. Fits were performed in a square region, of size ~ 2*σ_xy_, around the updated starting point. The σ_xy_ defines the size of 2D Gaussian approximation to the point spread function. After convergence of the fitting routine (a change in location of fewer than 10^-5^ pixels), a normalized cross-correlation was calculated to verify the 2D Gaussian-fitted coordinates. The found coordinates were only considered as positions of FN-IgG-EGFR and used in the further analysis if they exceeded a cross-correlation value of 0.7.

**3. Building trajectories from coordinates.** The probability of finding a diffusing particle with diffusivity *D* in two dimensions at a distance greater than *d* from its starting point after a time *Δt* is given by[^8^](#_ENREF_8)

$P\left( d,\Delta t \right)=exp\left[ \frac{-d^{2}}{4D\Delta t} \right]$ (1)

Trajectories were built from the set of 3D coordinates (x,y, and t) in two steps. First, coordinates identified at time *t* were compared with coordinates at time *t+Δt* using Eq. 1 where *Δt* is the inverse frame rate of data acquisition. If *P(r, Δt)* was found to be greater than .05, the coordinate at *t+Δt* is associated with the coordinate at *t* in a trajectory. This process builds short, un-interrupted trajectories. Second, to connect these short trajectories originated from the same targets, the end coordinate of all trajectories was compared with all later starting coordinates of other trajectories using Eq. 1, where *Δt* is now the time interval between the end of the first trajectory and the beginning of the second. The later trajectory with the smallest *Δt* that has a *P(r, Δt )*> 0.01 is connected with the first trajectory. This process is continued until there are no remaining pairs of trajectories that satisfy the criteria. The reconstructed trajectories are further processed into mean-squared displacement to estimate diffusion coefficient.

## **Method S5 | Extracting dynamic parameters from MSD**

The typical approach to analyze a single-particle trajectory starts with the calculation of mean-squared displacement (MSD)[^9^](#_ENREF_9)^,^ [^10^](#_ENREF_10), which describes the average squared distance (*d^2^, r* is the position vector) that the particle has explored in space at a given time lag (*Δt*):

$MSD\left( \Delta t \right)=\left\langle{(\boldsymbol{r}\left( t+\Delta t \right)-\boldsymbol{r}(t))}^{2} \right\rangle$ (2)

Similar to many groups[^11^](#_ENREF_11)^,^ [^12^](#_ENREF_12), we have observed that EGFR movement exhibited hop diffusion on the plasma membrane[^5^](#_ENREF_5). The dynamics parameters, the diffusivity (*D*) and the linear dimension of compartments (*L*), are extracted from trajectories by fitting the MSD curves. The *D* was defined as the linear MSD fitting result of the first 5 MSD points, and in the short time domain, the EGFR movement would act like Brownian motion:

$MSD\left( \Delta t \right)=\left\langle{(\boldsymbol{r}\left( t+\Delta t \right)-\boldsymbol{r}(t))}^{2} \right\rangle= 4D_{1-5}\Delta t+\sigma_{xy}^{2}$ (3)

The offset, $\sigma_{xy}^{2}$, specifies the localization precision σ_xy_ which was estimated using trajectories of fixed nanoparticles. The localization precision of our system is 7.3 ± 3.0 nm (mean ± standard deviation).

For *L*, the MSD curves were fitted with an equation for confined diffusion[^13^](#_ENREF_13)^,^ [^14^](#_ENREF_14), and *L* was defined as the MSD fitting result of the first 10 MSD points :

$MSD\left( \Delta t \right)\cong\frac{L^{2}}{3}\left[ 1-\exp\left( -\frac{\Delta t}{\tau} \right) \right]+4D_{macro}\Delta t+ \sigma_{xy}^{2}$ (4)

Confined diffusion is featured by an abrupt change of slope in the MSD curve[^11^](#_ENREF_11) after a characteristic equilibration time *τ.* In **Figure 1D**, the averaged MSD curves are down-ward curves within 0.5 second time window, which indicates the EGFRs exhibited confined diffusion. Therefore, we are able to extract the linear dimension of compartments (*L*).

## **Method S6 | Calculation of luminal differentiation score**

The luminal differentiation predictor was developed by Perou group[^15^](#_ENREF_15) and trained with the database GSE16997 which is contributed by Partanen group[^16^](#_ENREF_16). Then this predictor was applied to the UNC337 database (GSE18229 in GEO) to calculate the luminal differentiation scores of 39 tumor samples (n = 337). According to the molecular characterization, these 39 tumor samples have been classified into the 5 subtypes of breast cancer: Luminal A, Luminal B, HER2-enriched, Basal, and Claudin-low. Using gene expression profiling, Partanen group identified three epithelial cell-enriched subpopulations in the GSE16997 database: mammary stem cells (MaSC), luminal progenitors (pL) and mature luminal cells (mL). On the basis of the gene expression pattern of GSE16997 and these three classified subpopulations, the luminal differentiation predictor uses Distance-Weighted Discrimination (DWD)[^17^](#_ENREF_17), a type of High Dimension Low Sample Size statistical analysis methods, to define the separating hyperplane (with greatest variation between classified groups) and project the vectors of gene expression levels of tumor samples to the differentiation axes: MaSC⟶pL and pL⟶mL. In the training dataset (GSE16997), the pL centroid was set as the origin, and the MaSC and mL centroid were transformed to length 1 (sum of squares equals 1). After completing the differentiation model, each sample from UNC337 was then projected to the MaSC⟶pL axis and the pL⟶mL axis by calculating the inner product of the sample vector and the MaSC⟶pL vector or the pL⟶mL vector that was identified by DWD. The difference of the two projected positions of each sample along the MaSC⟶ pL⟶mL axis is defined as the differentiation score.

## **Method S7 | Immunofluorescence and structured illumination microscopy**

Cells were grown on an optical imaging 8-well chambered coverglass (154534, Thermo Scientific), fixed with 4% formaldehyde (F8775, Sigma-Aldrich), and permeabilized with 0.1% Triton X-100/PBS (Triton X-100, T8787, Sigma-Aldrich) prior to blocking with 1% BSA in PBS. Then, the samples were incubated with antibodies overnight at 4ºC. To assess EMT status, cells were characterized with an EMT immunochemistry kit (SC026, R&D systems) which contains three types of fluorophore-tagged IgGs: NL557-conjugated goat anti-human snail, NL637-conjugated goat anti-human E-cadherin, and NL493-conjugated goat anti-human vimentin. Cell nuclei were visualized with Hoechst 33258 (H3569, Thermo Fisher Scientific). The Alexa Fluor^TM^ 633 Phalloidin (A22284, ThermoFisher Scientific) was used to stain actin filaments (F-actin). The fluorescence imaging was taken by Elyra S.1 Structured Illumination Super-Resolution Microscope (SR-SIM) with a 63x 1.2 N.A. water objective. The SR-SIM system is equipped with 4 excitation laser wavelengths for standard fluorophores (405 nm, 488nm, 561 nm, and 633nm) and comes with emission filters for DAPI, GFP, RFP and Alexa 633.

## **Method S8 | Derivation of arithmetic moments from normally distributed log *D* and log *L***

The arithmetic mean ($\bar{X}$) and arithmetic standard deviation ($\sigma_{\bar{x}}$) shown in Figure 4 and Figure S2 were derived as follows. Given a log-normally distributed random variable *X* and two parameters *µ* and *σ*, which are, respectively, the mean and standard deviation of the variable’s logarithm, then the logarithm of *X* is normally distributed, and we can write *X* as

$X={10}^{\mu+\sigma Z}$ (5)

with Z a standard normal variable[^18^](#_ENREF_18). Then the arithmetic mean and arithmetic standard deviation of the log-normally distributed *X* are given by[^18^](#_ENREF_18)

$\bar{X}=E\left[ X \right]={10}^{\mu+0.5\sigma^{2}}$ (6)

$E\left[ X^{2} \right]={10}^{2\mu+2\sigma^{2}}$ (7)

$Var\left[ X \right]=E\left[ X^{2} \right]-\left[ E\left[ X \right] \right]^{2}={10}^{2\mu+\sigma^{2}}({10}^{\sigma^{2}}-1)$ (8)

$\sigma_{\bar{x}}=SD\left[ X \right]=\sqrt{Var[X]}=E\left[ X \right]\sqrt{{10}^{\sigma^{2}}-1}={10}^{\mu+0.5\sigma^{2}}\sqrt{{10}^{\sigma^{2}}-1}$ (9)

## **Method S9 | Homogenous time-resolved fluorescence (HTRF)**

Cells were seeded in 96-well culture plates with the seeding density of 20,000 cells/well. After 2-day incubation followed 1-day serum starvation, the cells were stimulated with 20 ng/mL EGF (recombinant human epidermal growth factor, PHG0311L, Thermo Fisher Scientific) in serum-free DMEM/F12 medium and subsequently lysed at a series of time points (0, 15, 30, 60, 120, 300 seconds). For EMT-induced cells, the cells were treated with the EMT-induction supplement for 3 days before being transferred in 96-well culture plates. The EMT-induced cells were incubated in the medium containing the EMT-induction supplement for 2-day and then 1-day starvation. The cellular lysates were transferred to two assay plates for Phospho-EGFR (Tyr1068) Cellular Assay Kit (64EG1PEG, cisbio) and Total EGFR Cellular Assay Kit (64NG1PEG, cisbio). Anti-phospho-EGFR-d2 (2µl) and anti-EGFR-Eu^3+^-Cryptate (2µl) were added to each well and plates were incubated in the dark for 4h at room temperature. The HTRF signals were read by Flexstation® 3 of Molecular Devices, and the measurement conditions were listed below: 50 µs integration delay, 400 µs integration, 100 reading, 314 nm wavelength for both excitation 1 and 2, 665 nm for emission 1, 630 nm for cut off of emission 1, 620 nm for emission 2, and 570 nm for cut off of emission 2. The HTRF ratio, background corrected HTRF ratio, and the normalized value was calculated as

$HTRF Ratio=\frac{Signal 665 nm}{Signal 620 nm}\times{10}^{4}$ (10)

$$\Delta R=Background corrected HTRF Ratio$$

$={HTRF Ratio}_{sample}-{HTRF Ratio}_{background}$ (11)

$Normalized \Delta R=\frac{{\Delta R}_{Phospho-EGFR}}{{\Delta R}_{Total-EGFR}}\times100$ (12)

Levels of phosphorylated EGFR at different time points were further normalized to those obtained at 0s for each experimental group. Each data point has four repeats, and we have conducted three trials.


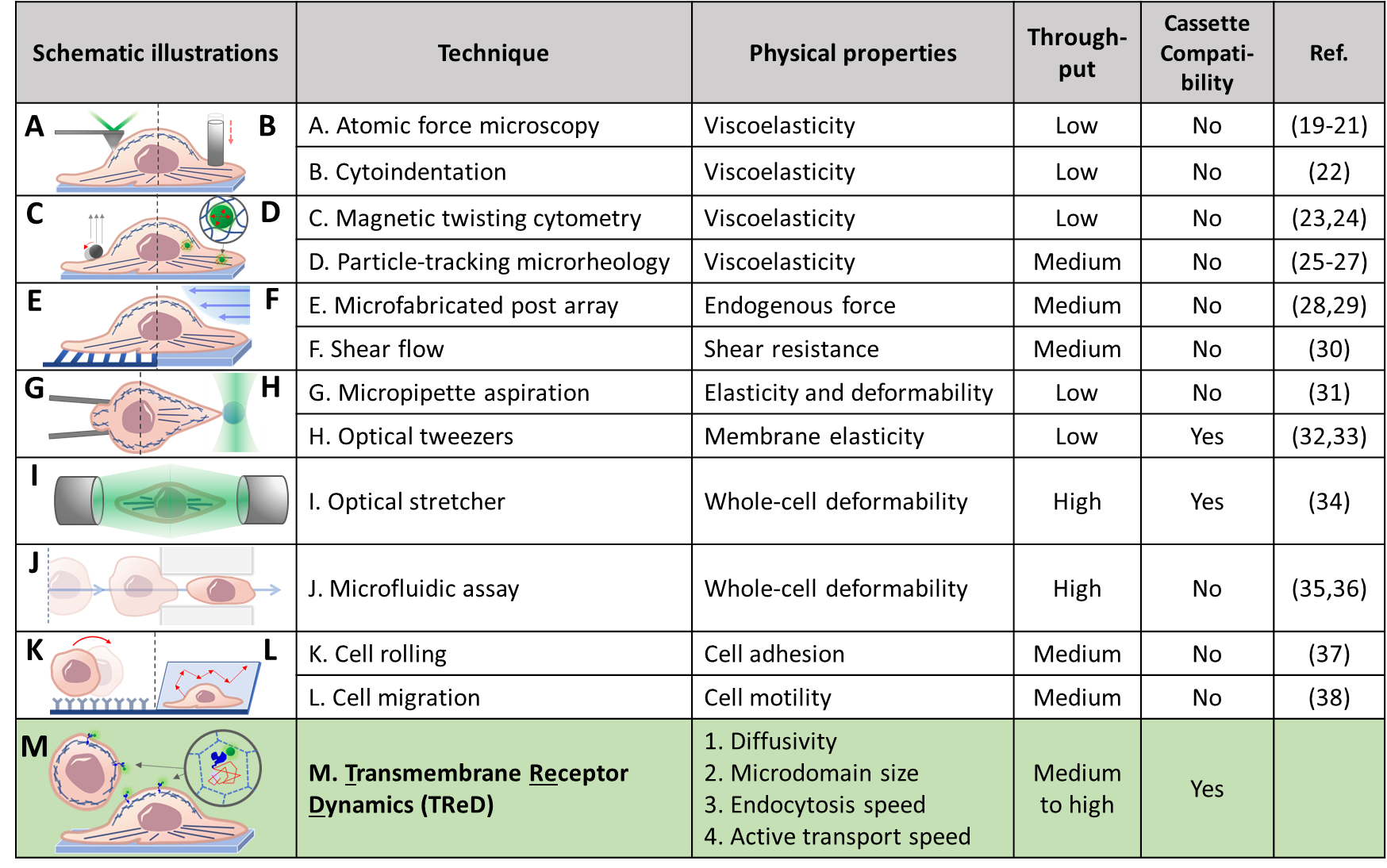


## **Figure S1 | Assays for biophysical characterization of cells**

**Fig. S1** summarized a variety of techniques for conducting physical or mechanical phenotyping of cells in term of principles, physical properties to be measured, throughput, and the cassette compatibility. AFM-based methods[^19^](#_ENREF_19)^,^ [^20^](#_ENREF_20)^,^ [^21^](#_ENREF_21), cytoindentation[^22^](#_ENREF_22), and magnetic twisting cytometry[^23^](#_ENREF_23)^,^ [^24^](#_ENREF_24) exert forces on a local region of an adherent cell. Therefore, these techniques suffer from low throughput, and the direct contact of probes with the cell surface may cause cellular responses. Without applied forces does particle-tracking microrheology[^25^](#_ENREF_25)^,^ [^26^](#_ENREF_26)^,^ [^27^](#_ENREF_27) allow the measurement of intracellular viscoelasticity, but the ballistic injection or micro-injection of nanoparticles into cells requires significant time and might jeopardize cell viability. Cell traction force[^28^](#_ENREF_28)^,^ [^29^](#_ENREF_29) and shear stress resistance[^30^](#_ENREF_30) which measured by microfabricated post array and shear flow technique, respectively, could also provide a better understanding of cancer cell metastasis. However, these six techniques we discussed above can be used only for cells adhered on a substrate, which would constrain the throughput and might limit their applicability of being integrated into BioMEMS-based circulating tumor cell (CTC) capture devices. Micropipette aspiration[^31^](#_ENREF_31) has modest throughput and can deal with suspended cells, but it also can’t avoid cell-tool interactions. Light-based cell-manipulation approaches, including optical tweezers[^32^](#_ENREF_32)^,^ [^33^](#_ENREF_33) and optical stretcher[^34^](#_ENREF_34), are attractive methods that do not require mechanical contact with the cells under examination. However, one the limitations of using optical tweezers and optical stretcher for measuring the whole-cell deformability is the relatively small-magnitude force (10^-9^-10^-14^ N) that can be applied. The force is not strong enough to deform most cell types sufficiently to obtain information about whole-cell properties. Microfluidic assays[^35^](#_ENREF_35)^,^ [^36^](#_ENREF_36), such as deformation passage and hydrodynamic stretching, provide high-throughput mechanical phenotyping of whole cells under precisely controlled fluid flow. The microfluidic channels need to be properly designed to avoid the induced mechanical stress on cells. The cell rolling[^37^](#_ENREF_37) and the cell migration[^38^](#_ENREF_38), only provide medium throughput. Our technique is an innovative approach to measure the physical properties of cells by tracking fluorescence-tagged transmembrane receptors. This technique can be easily integrated with a microfluidic CTC capture device which exhibits the high-throughput isolation of CTCs from blood (**Fig. S3**); this technique can deal with suspended cells and does not require mechanical contact or applied force with cells under examination. In addition, the acquired trajectories from several subcellular regions in one single cell (5-20 trajectories) would provide the higher spatial resolution than those from whole-cell measurements.


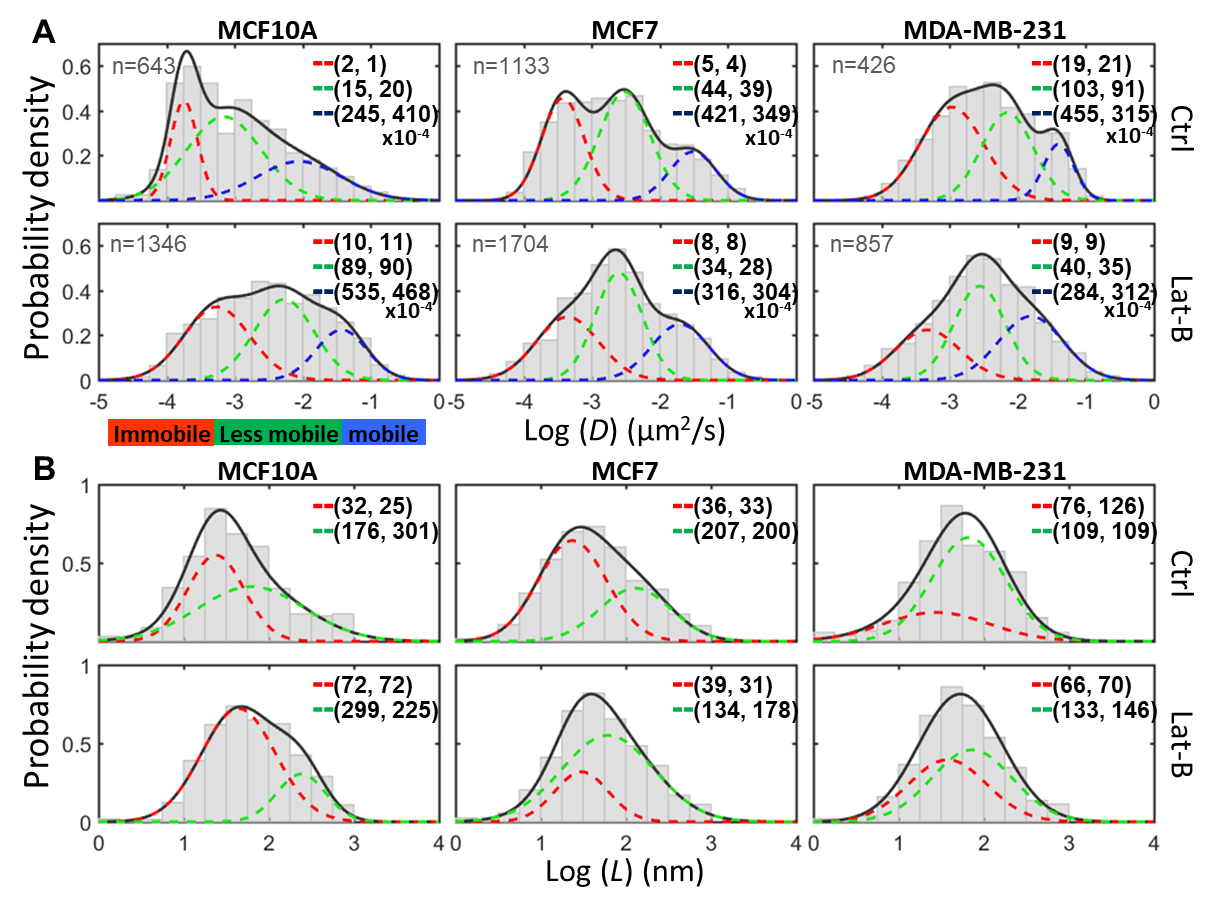


## **Figure S2 | LatB changes the diffusivity of EGFR and the size of microdomains.**

Histograms of log *D* (**A**) and log *L* (**B**) extracted from FN-IgG-EGFRs trajectories in the three breast cell lines that were treated with/without 200 nM latrunculin B (LatB). The values shown in the histograms represent the arithmetic moments of *D* or *L* ((arithmetic means ($\bar{X}$) , arithmetic strandard deviations($\sigma_{\bar{x}}$) )) derived from Gaussian mixture model fitting of log *D* and log *L* (3 or 2 sub-populations). The three populations are defined as immobilization (red), less mobile (green), and mobile (blue). The number of trajectories from each group is labeled in (A).


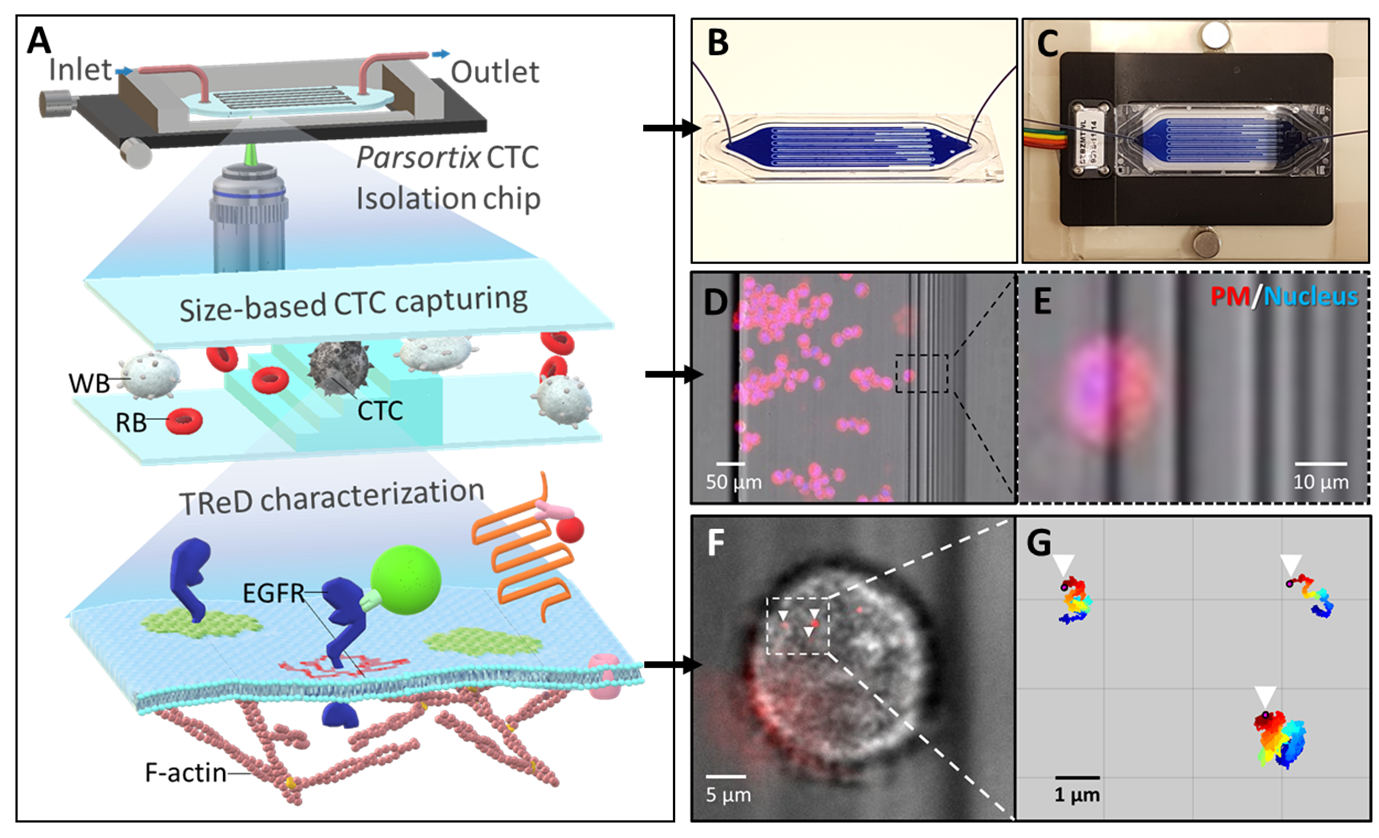


## **Figure S3 | Cassette compatibility of TReD assay**

**(A)** The schematic shows the procedure of TReD assay applied in a cell-capturing microfluidic chip. We demonstrated the cassette compatibility on the Parsortix CTC isolation chip which uses the size-based cell capture technique to isolate cancer cells. Being trapped in the microfluidic chip, cells were tagged with FN-IgG and followed with SPT of FN-IgG-EGFR. (**B**) The Parsotix CTC isolation chip filled with blue ink. (**C**) The chip can be mounted on the temperature-controlled stage during tracking. (**D**) We use MDA-MB-231 as the substitute of CTCs to test the cassette compatibility of the TReD assay. The plasma membrane (PM) and nuclei were stained with CellMask-Deep Red and Hoechst, respectively. (**E**) The zoom-in shows a captured cell. **(F)** The EGFRs were tagged with FN-IgGs (red-colored and pointed by arrowheads) (**G**) The zoom-in shows the collected trajectories from a captured cell.


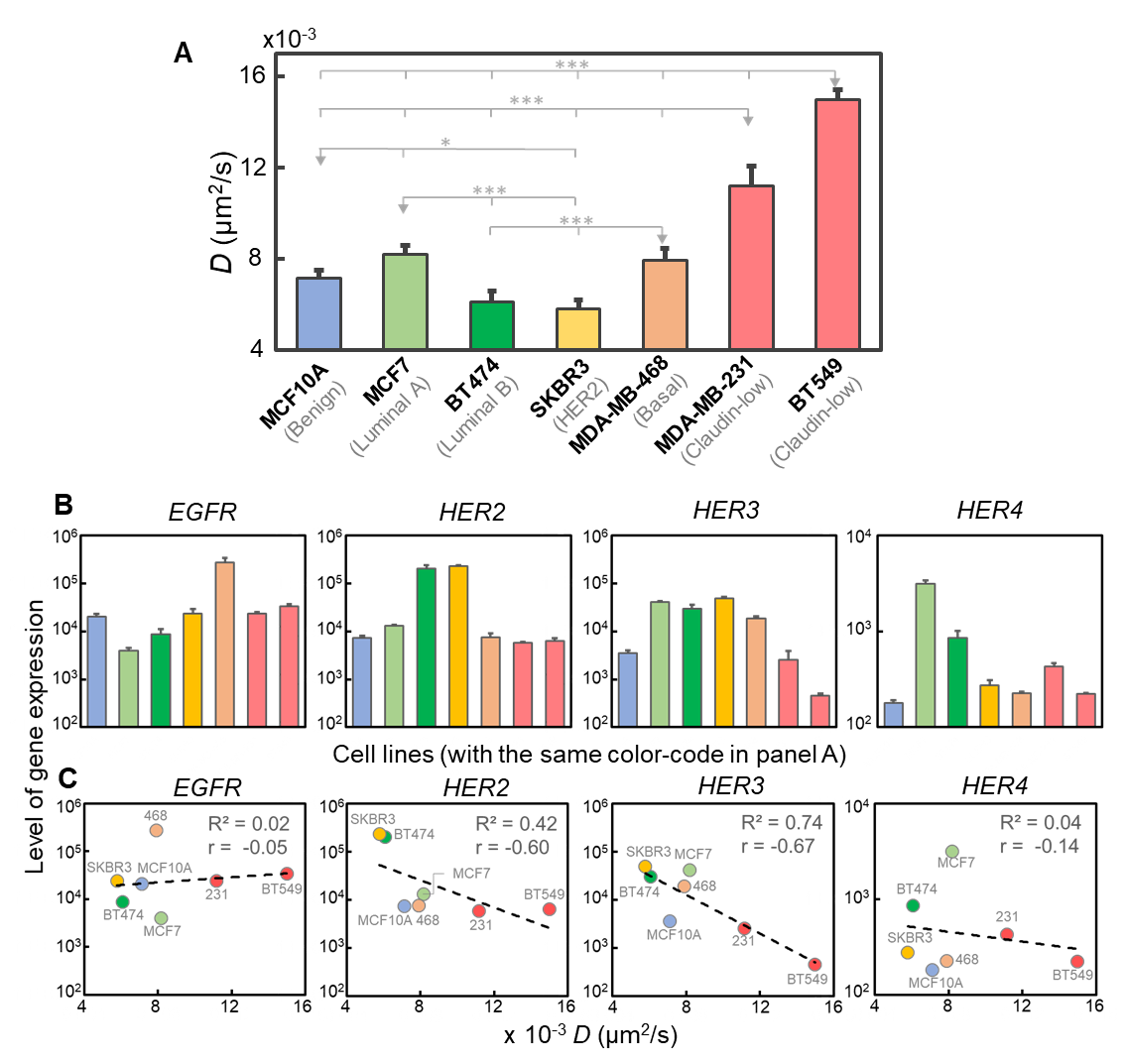


## **Figure S4 | Correlations between EGFR diffusivity and expression levels of EGFR family**

**(A)** The diffusivity of EGFR complexes measured in these seven breast cell lines. The error bar represents the standard error. (**B**) mRNA expression levels of the EGFR family including *EGFR*, *HER2*, *HER3*, and *HER4* among these cells lines. Each bar is color-coded with the same color corresponding to each type of cells shown in panel A. (**C**) Correlations between expression levels of receptors to EGFR diffusivity. The R-squared is calculated by exponential curve fitting, and the r represents the correlation coefficient.

## **Table S1 | Molecular classification, clinical and pathological features, and sources of cell lines**


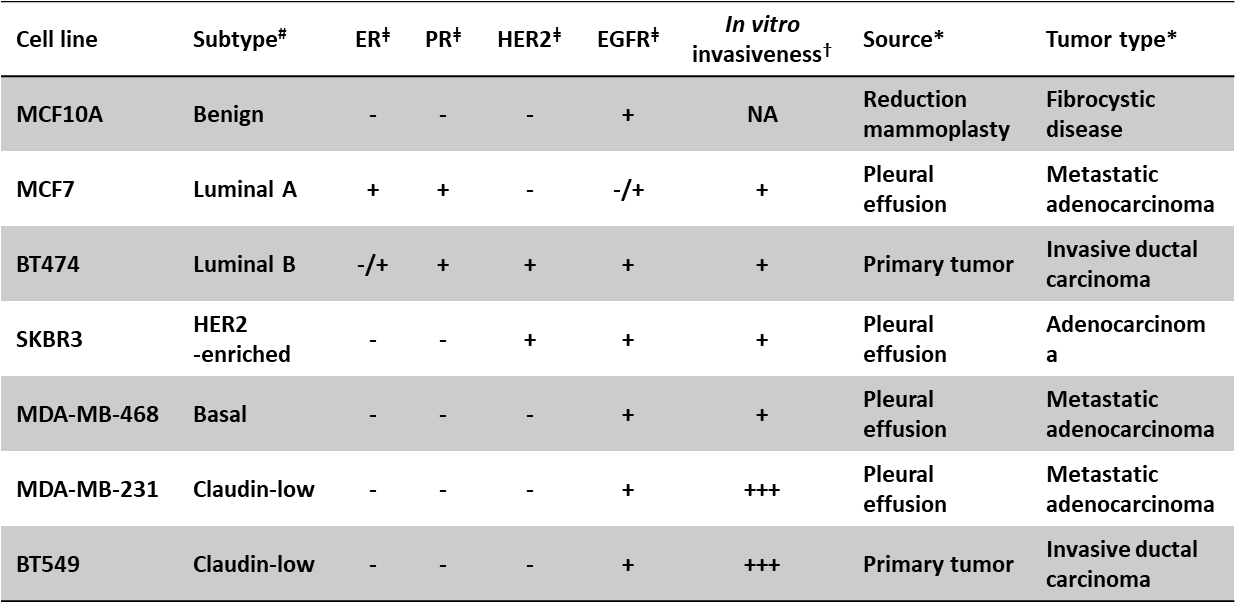


Abbreviations: ER, estrogen receptor; PR, progesterone receptor; HER2, human epidermal growth factor receptor 2; EGFR, epidermal growth factor receptor.

^#^ The five subtypes are determined from Neve’s[^39^](#_ENREF_39) and Prat’s[^40^](#_ENREF_40) study.

ǂ The biomolecular characteristics are determined from Neve’s[^39^](#_ENREF_39), Kao’s[^41^](#_ENREF_41), and Subik’s[^42^](#_ENREF_42) study.

^†^ The levels of *in vitro* invasiveness are determined from Oberst’s study[^43^](#_ENREF_43).

*The information of source and tumor type is from the ATCC (<https://www.atcc.org/>)

## **Table S2 | Source of gene expression data (mRNA microarray)**

| **Cell line** | **Source of gene expression data** |
| --- | --- |
| MCF10A | GSE5823[^44^](#_ENREF_44), GSE10070[^45^](#_ENREF_45), GSE34211[^46^](#_ENREF_46), E-MEXP-3682[^47^](#_ENREF_47), E-MEXP-3407[^48^](#_ENREF_48) |
| MCF7 | GSE5823[^44^](#_ENREF_44), GSE6521, GSE8597[^49^](#_ENREF_49), GSE10879, GSE11135[^50^](#_ENREF_50), GSE11324[^51^](#_ENREF_51), GSE11352[^52^](#_ENREF_52), GSE11506[^53^](#_ENREF_53), GSE11791[^54^](#_ENREF_54), GSE13477[^55^](#_ENREF_55), GSE19123[^56^](#_ENREF_56), GSE36133[^57^](#_ENREF_57), GSE34211[^46^](#_ENREF_46), E-MTAB-37[^58^](#_ENREF_58), GSE46924[^59^](#_ENREF_59), GSE57083, GSE32474[^60^](#_ENREF_60), GSE31912[^61^](#_ENREF_61), GSE36847[^62^](#_ENREF_62), GSE29884 |
| BT474 | GSE5823[^44^](#_ENREF_44), GSE36133[^57^](#_ENREF_57), GSE34211[^46^](#_ENREF_46), E-MTAB-37[^58^](#_ENREF_58), GSE57083 |
| SKBR3 | GSE7562[^63^](#_ENREF_63), GSE36133[^57^](#_ENREF_57), GSE34211[^46^](#_ENREF_46), GSE57083 |
| MDA-MB-468 | GSE36133[^57^](#_ENREF_57), GSE57083 |
| MDA-MB-231 | GSE5823[^44^](#_ENREF_44), GSE7307, GSE20089[^64^](#_ENREF_64), GSE36133[^57^](#_ENREF_57), GSE34211[^46^](#_ENREF_46),  GSE15893[^65^](#_ENREF_65), GSE57083, GSE32474[^60^](#_ENREF_60) |
| BT549 | GSE36133[^57^](#_ENREF_57), GSE34211[^46^](#_ENREF_46), GSE57083, GSE32474[^60^](#_ENREF_60) |

## **Table S3 | Selected genes for gene expression analysis**


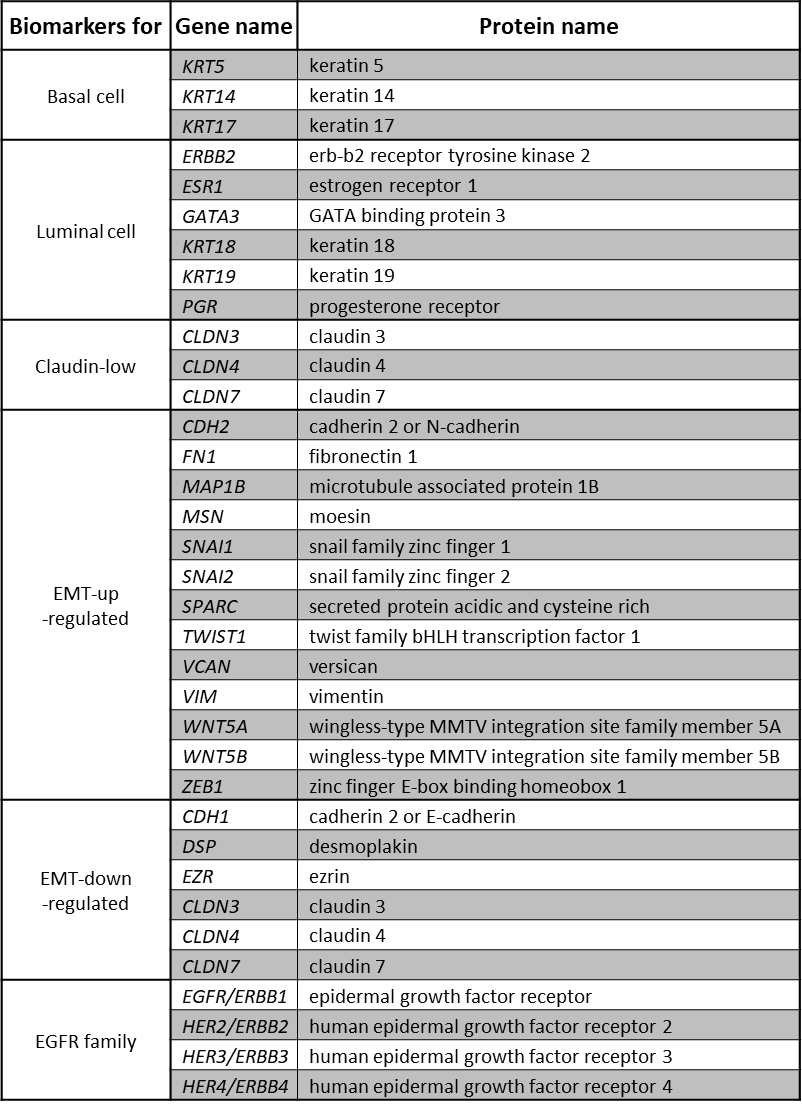


## **Supplementary References**

1. Debnath J, Muthuswamy SK, Brugge JS. Morphogenesis and oncogenesis of MCF-10A mammary epithelial acini grown in three-dimensional basement membrane cultures. *Methods* **30**, 256-268 (2003).

2. Pons T, Uyeda HT, Medintz IL, Mattoussi H. Hydrodynamic dimensions, electrophoretic mobility, and stability of hydrophilic quantum dots. *The Journal of Physical Chemistry B* **110**, 20308-20316 (2006).

3. Hendriks CL, Van Vliet L, Rieger B, van Kempen G, van Ginkel M. DIPimage: a scientific image processing toolbox for MATLAB. *Quantitative Imaging Group, Faculty of Applied Sciences, Delft University of Technology, Delft, The Netherlands*, (1999).

4. Andrews NL*, et al.* Actin restricts Fc&epsiv; RI diffusion and facilitates antigen-induced receptor immobilization. *Nature cell biology* **10**, 955-963 (2008).

5. Liu Y-L*, et al.* Segmentation of 3D Trajectories Acquired by TSUNAMI Microscope: An Application to EGFR Trafficking. *Biophysical Journal* **111**, 2214-2227 (2016).

6. Arndt-Jovin DJ*, et al.* In vivo cell imaging with semiconductor quantum dots and noble metal nanodots. In: *Colloidal Quantum Dots for Biomedical Applications* (ed^(eds). International Society for Optics and Photonics (2006).

7. Dahan M, Levi S, Luccardini C, Rostaing P, Riveau B, Triller A. Diffusion dynamics of glycine receptors revealed by single-quantum dot tracking. *Science* **302**, 442-445 (2003).

8. Saxton MJ. Lateral Diffusion in an Archipelago - Single-Particle Diffusion. *Biophysical Journal* **64**, 1766-1780 (1993).

9. Martin DS, Forstner MB, Käs JA. Apparent subdiffusion inherent to single particle tracking. *Biophysical Journal* **83**, 2109-2117 (2002).

10. Saxton MJ, Jacobson K. Single-particle tracking: applications to membrane dynamics. *Annual Review of Biophysics and Biomolecular Structure* **26**, 373-399 (1997).

11. Kusumi A, Sako Y, Yamamoto M. Confined lateral diffusion of membrane receptors as studied by single particle tracking (nanovid microscopy). Effects of calcium-induced differentiation in cultured epithelial cells. *Biophysical journal* **65**, 2021-2040 (1993).

12. Clausen MP, Lagerholm BC. Visualization of plasma membrane compartmentalization by high-speed quantum dot tracking. *Nano letters* **13**, 2332-2337 (2013).

13. Daumas F, Destainville N, Millot C, Lopez A, Dean D, Salome L. Confined diffusion without fences of a G-protein-coupled receptor as revealed by single particle tracking. *Biophysical Journal* **84**, 356-366 (2003).

14. Di Rienzo C, Gratton E, Beltram F, Cardarelli F. Fast spatiotemporal correlation spectroscopy to determine protein lateral diffusion laws in live cell membranes. *Proceedings of the National Academy of Sciences* **110**, 12307-12312 (2013).

15. Prat A*, et al.* Phenotypic and molecular characterization of the claudin-low intrinsic subtype of breast cancer. *Breast cancer research* **12**, R68 (2010).

16. Lim E*, et al.* Aberrant luminal progenitors as the candidate target population for basal tumor development in BRCA1 mutation carriers. *Nature medicine* **15**, 907 (2009).

17. Marron JS, Todd MJ, Ahn J. Distance-weighted discrimination. *Journal of the American Statistical Association* **102**, 1267-1271 (2007).

18. Johnson NL, Kotz S, Balakrishnan N. Lognormal distributions. *Continuous univariate distributions* **1**, 207-227 (1994).

19. Osmulski P*, et al.* Nanomechanical biomarkers of single circulating tumor cells for detection of castration resistant prostate cancer. *The Prostate* **74**, 1297-1307 (2014).

20. Radmacher M, Fritz M, Kacher CM, Cleveland JP, Hansma PK. Measuring the viscoelastic properties of human platelets with the atomic force microscope. *Biophysical journal* **70**, 556-567 (1996).

21. Calzado-Martín A, Encinar M, Tamayo J, Calleja M, San Paulo A. Effect of actin organization on the stiffness of living breast cancer cells revealed by peak-force modulation atomic force microscopy. *ACS nano* **10**, 3365-3374 (2016).

22. Shin D, Athanasiou K. Cytoindentation for obtaining cell biomechanical properties. *Journal of Orthopaedic Research* **17**, 880-890 (1999).

23. Puig-de-Morales-Marinkovic M, Turner KT, Butler JP, Fredberg JJ, Suresh S. Viscoelasticity of the human red blood cell. *American Journal of Physiology-Cell Physiology* **293**, C597-C605 (2007).

24. Wang N, Ingber DE. Probing transmembrane mechanical coupling and cytomechanics using magnetic twisting cytometry. *Biochemistry and Cell Biology* **73**, 327-335 (1995).

25. Mason T, Ganesan K, Van Zanten J, Wirtz D, Kuo S. Particle tracking microrheology of complex fluids. *Physical Review Letters* **79**, 3282 (1997).

26. Guo M*, et al.* Probing the stochastic, motor-driven properties of the cytoplasm using force spectrum microscopy. *Cell* **158**, 822-832 (2014).

27. Wirtz D. Particle-tracking microrheology of living cells: principles and applications. *Annual review of biophysics* **38**, 301-326 (2009).

28. Tan JL, Tien J, Pirone DM, Gray DS, Bhadriraju K, Chen CS. Cells lying on a bed of microneedles: an approach to isolate mechanical force. *Proceedings of the National Academy of Sciences* **100**, 1484-1489 (2003).

29. Paszek MJ*, et al.* Tensional homeostasis and the malignant phenotype. *Cancer cell* **8**, 241-254 (2005).

30. Bao G, Suresh S. Cell and molecular mechanics of biological materials. *Nature materials* **2**, 715 (2003).

31. Hochmuth RM. Micropipette aspiration of living cells. *Journal of biomechanics* **33**, 15-22 (2000).

32. Dao M, Lim CT, Suresh S. Mechanics of the human red blood cell deformed by optical tweezers. *Journal of the Mechanics and Physics of Solids* **51**, 2259-2280 (2003).

33. Zhang H, Liu K-K. Optical tweezers for single cells. *Journal of The Royal Society Interface* **5**, 671-690 (2008).

34. Guck J*, et al.* Optical deformability as an inherent cell marker for testing malignant transformation and metastatic competence. *Biophysical journal* **88**, 3689-3698 (2005).

35. Hou HW, Li Q, Lee G, Kumar A, Ong C, Lim CT. Deformability study of breast cancer cells using microfluidics. *Biomedical microdevices* **11**, 557-564 (2009).

36. Gossett DR*, et al.* Hydrodynamic stretching of single cells for large population mechanical phenotyping. *Proceedings of the National Academy of Sciences* **109**, 7630-7635 (2012).

37. Network TPS-OC. A physical sciences network characterization of non-tumorigenic and metastatic cells. *Scientific reports* **3**, (2013).

38. Kramer N*, et al.* In vitro cell migration and invasion assays. *Mutation Research/Reviews in Mutation Research* **752**, 10-24 (2013).

39. Neve RM*, et al.* A collection of breast cancer cell lines for the study of functionally distinct cancer subtypes. *Cancer cell* **10**, 515-527 (2006).

40. Prat A, Perou CM. Deconstructing the molecular portraits of breast cancer. *Molecular oncology* **5**, 5-23 (2011).

41. Kao J*, et al.* Molecular profiling of breast cancer cell lines defines relevant tumor models and provides a resource for cancer gene discovery. *PloS one* **4**, e6146 (2009).

42. Subik K*, et al.* The expression patterns of ER, PR, HER2, CK5/6, EGFR, Ki-67 and AR by immunohistochemical analysis in breast cancer cell lines. *Breast cancer: basic and clinical research* **4**, 35 (2010).

43. Oberst M*, et al.* Matriptase and HAI-1 are expressed by normal and malignant epithelial cells in vitro and in vivo. *The American journal of pathology* **158**, 1301-1311 (2001).

44. Cappellen D, Schlange T, Bauer M, Maurer F, Hynes NE. Novel c-MYC target genes mediate differential effects on cell proliferation and migration. *EMBO Rep* **8**, 70-76 (2007).

45. Marshall AM, Pai VP, Sartor MA, Horseman ND. In vitro multipotent differentiation and barrier function of a human mammary epithelium. *Cell Tissue Res* **335**, 383-395 (2009).

46. Hook KE*, et al.* An integrated genomic approach to identify predictive biomarkers of response to the aurora kinase inhibitor PF-03814735. *Mol Cancer Ther* **11**, 710-719 (2012).

47. Odrowaz Z, Sharrocks AD. The ETS transcription factors ELK1 and GABPA regulate different gene networks to control MCF10A breast epithelial cell migration. *PLoS One* **7**, e49892 (2012).

48. Odrowaz Z, Sharrocks AD. ELK1 uses different DNA binding modes to regulate functionally distinct classes of target genes. *PLoS genetics* **8**, e1002694 (2012).

49. Bourdeau V, Deschenes J, Laperriere D, Aid M, White JH, Mader S. Mechanisms of primary and secondary estrogen target gene regulation in breast cancer cells. *Nucleic Acids Res* **36**, 76-93 (2008).

50. Kohlmann A*, et al.* An international standardization programme towards the application of gene expression profiling in routine leukaemia diagnostics: the Microarray Innovations in LEukemia study prephase. *British journal of haematology* **142**, 802-807 (2008).

51. Carroll JS*, et al.* Genome-wide analysis of estrogen receptor binding sites. *Nature genetics* **38**, 1289-1297 (2006).

52. Lin CY*, et al.* Whole-genome cartography of estrogen receptor alpha binding sites. *PLoS genetics* **3**, e87 (2007).

53. Lin Z, Reierstad S, Huang CC, Bulun SE. Novel estrogen receptor-alpha binding sites and estradiol target genes identified by chromatin immunoprecipitation cloning in breast cancer. *Cancer Res* **67**, 5017-5024 (2007).

54. Musgrove EA*, et al.* Identification of functional networks of estrogen- and c-Myc-responsive genes and their relationship to response to tamoxifen therapy in breast cancer. *PLoS One* **3**, e2987 (2008).

55. Stockwin LH, Yu SX, Stotler H, Hollingshead MG, Newton DL. ARC (NSC 188491) has identical activity to Sangivamycin (NSC 65346) including inhibition of both P-TEFb and PKC. *Bmc Cancer* **9**, 63 (2009).

56. Chen JL*, et al.* Lactic acidosis triggers starvation response with paradoxical induction of TXNIP through MondoA. *PLoS genetics* **6**, e1001093 (2010).

57. Barretina J*, et al.* The Cancer Cell Line Encyclopedia enables predictive modelling of anticancer drug sensitivity. *Nature* **483**, 603-607 (2012).

58. Krupp M, Itzel T, Maass T, Hildebrandt A, Galle PR, Teufel A. CellLineNavigator: a workbench for cancer cell line analysis. *Nucleic Acids Res* **41**, D942-948 (2013).

59. Nelson ER*, et al.* 27-Hydroxycholesterol links hypercholesterolemia and breast cancer pathophysiology. *Science* **342**, 1094-1098 (2013).

60. Pfister TD*, et al.* Topoisomerase I levels in the NCI-60 cancer cell line panel determined by validated ELISA and microarray analysis and correlation with indenoisoquinoline sensitivity. *Mol Cancer Ther* **8**, 1878-1884 (2009).

61. Wang L*, et al.* Cell cycle gene networks are associated with melanoma prognosis. *PLoS One* **7**, e34247 (2012).

62. Larsson O*, et al.* Distinct perturbation of the translatome by the antidiabetic drug metformin. *Proceedings of the National Academy of Sciences of the United States of America* **109**, 8977-8982 (2012).

63. Vivanco I*, et al.* Identification of the JNK signaling pathway as a functional target of the tumor suppressor PTEN. *Cancer cell* **11**, 555-569 (2007).

64. Shankar J, Messenberg A, Chan J, Underhill TM, Foster LJ, Nabi IR. Pseudopodial actin dynamics control epithelial-mesenchymal transition in metastatic cancer cells. *Cancer Res* **70**, 3780-3790 (2010).

65. Appaiah H, Bhat-Nakshatri P, Mehta R, Thorat M, Badve S, Nakshatri H. ITF2 is a target of CXCR4 in MDA-MB-231 breast cancer cells and is associated with reduced survival in estrogen receptor-negative breast cancer. *Cancer biology & therapy* **10**, 600-614 (2010).
